# The impact of data imputation on air quality prediction problem

**DOI:** 10.1371/journal.pone.0306303

**Published:** 2024-09-12

**Authors:** Van Hua, Thu Nguyen, Minh-Son Dao, Hien D. Nguyen, Binh T. Nguyen

**Affiliations:** 1 Faculty of Mathematics and Computer Science, University of Science, Ho Chi Minh City, Vietnam; 2 Vietnam National University Ho Chi Minh City, Ho Chi Minh City, Vietnam; 3 University of Information Technology, Ho Chi Minh City, Vietnam; 4 National Institute of Information and Communications Technology, Tokyo, Japan; 5 SimulaMet, Oslo, Norway; 6 Faculty of Information Technology, HUTECH University, Ho Chi Minh City, Vietnam; Islamia University of Bahawalpur, PAKISTAN

## Abstract

With rising environmental concerns, accurate air quality predictions have become paramount as they help in planning preventive measures and policies for potential health hazards and environmental problems caused by poor air quality. Most of the time, air quality data are time series data. However, due to various reasons, we often encounter missing values in datasets collected during data preparation and aggregation steps. The inability to analyze and handle missing data will significantly hinder the data analysis process. To address this issue, this paper offers an extensive review of air quality prediction and missing data imputation techniques for time series, particularly in relation to environmental challenges. In addition, we empirically assess eight imputation methods, including mean, median, kNNI, MICE, SAITS, BRITS, MRNN, and Transformer, to scrutinize their impact on air quality data. The evaluation is conducted using diverse air quality datasets gathered from numerous cities globally. Based on these evaluations, we offer practical recommendations for practitioners dealing with missing data in time series scenarios for environmental data.

## 1 Introduction

Air helps sustain human life, so air tracking and understanding its quality is essential for our health. Air pollutants can pose significant threats to public health, and sources of air pollution can come from nature, such as smoke from volcano eruptions or forest fires, methane from animals’ process of digesting food, or radon gas from radioactive decay in the earth’s crust. In addition, pollution can also come from manufacturing activities such as industry and agriculture. They emit *CO*_2_, *CO*, *SO*_2_, *NO*_2_, and other organic substances at extremely high concentrations, polluting the air. Besides, burning fossil fuels yields climate change and air pollution. Therefore, air quality has still been a concern in recent years. Consequently, environmental researchers mine air quality data to uncover potential value and information from these data, thereby capturing user behavior, estimating disease causes, discovering gases, detecting individual actions to reduce greenhouse gases, acid rain, etc., and then advising management agencies and local governments to plan related policies. By using machine learning techniques, the local air quality data can be analyzed using sensors that gather real-time humidity and temperature readings. Duong et al. (2021) effectively extracted pertinent features from a dataset. They applied machine learning models to forecast AQI (Air Quality Indexing) values and levels at any user-specified location in Ho Chi Minh City [1]. The dataset includes data on six atmospheric pollutants: *SO*_2_, *NO*_2_, *PM*10, *PM*2.5, *CO*, and *O*_3_, collected by volunteers who traversed predetermined routes to provide ground-truth AQI levels.

Many air quality data are in the form of time series and can contain missing values due to corrupted sensors, loss of electricity, etc. In such cases, data imputation, i.e., filling in missing values with some reasonable value according to some criteria, is a conventional practice to resolve the issue. The quality of imputation can significantly impact the downstream classification or prediction task. One can characterize missing data into three types: *missing completely at random* (MCAR), where the missing values are independent of any other values; *missing at random*(MAR), where missing values depend only on observed values; and *missing not at random*(MNAR), where missing values depends on both observed and unobserved values [2]. There are many methods to deal with missing values based on the missing data mechanism. This work focuses on the MAR case, as it is prevalent for sensor data related to the environment [3]. Furthermore, most air quality observation data are time series data. Dealing with missing values in time series data is often difficult, time-consuming, and labor-intensive. In addition, the missing data can significantly affect the processing and analysis of data. Therefore, handling missing values in time-series air quality data is necessary.

People can reveal critical enhancements regarding performance and running time by examining newly introduced approaches for data imputation. Multiple imputations can be further applied with these imputation methods to reduce the uncertainty by repeating the imputation procedure numerous times and averaging the results. Combining the imputation methods with forecasting models often results in a two-step process where imputation and forecasting models are separated. By doing this, the missingness is effectively explored in the forecasting model, thus leading to suboptimal analysis results. In addition, many imputation methods also have other requirements that may not be satisfied in real applications; for example, many of them work on data with low missing rates only, assume the data is missing randomly or completely at random, or can not handle time series data with varying lengths [4]. Moreover, training and applying these imputation methods are usually computationally expensive.

Various imputation techniques have been proposed to fill in missing values, each using a distinct set of assumptions, algorithms, and performance metrics. Choosing a relevant imputation method can significantly influence the subsequent analysis and the reliability of the results. To give a thorough comparative analysis of various missing data imputation methods for time series air quality of data [5], we compare several conventional but often used imputation techniques (mean, median, kNNI, and MICE) with several recently developed imputation techniques for temporal data (SAITS, BRITS, MRNN, and Transformer) to examine their impact on air quality data from various places. On the other hand, the rate of missing data can also impact the problem-solving strategy we use since missing values can be handled in the step of data preprocessing. Various works have conducted experiments under a variety of missing rates. For example, [6] conducts experiments with missing rates from 5% to 50%. Nevertheless, in some other papers, the missing values rate could range from 1% to 80% of data [7–9], or start from 10% to 50% [10].

While some work [11] has been done to compare the performance of classical and newly developed time series imputation techniques such as BRITS [12], SAITS [13] for health care data, such practical comparisons for air quality has not been conducted yet. In addition, while there have been several works that examine the effects of imputation on air quality [14–16], most of them do not cover state-of-the-art imputation methods for time series that have been developed in recent years. In addition, up to our knowledge, while there have been some surveys on air quality prediction [17, 18] or missing data imputation for air quality data [19, 20], there has not been any work that reviews both problems and systematically compares state-of-the-art imputation algorithms for air quality data. This motivates us to review recent studies related to the air quality prediction problem, along with missing values handling methods or techniques on time series data with a concentration on air quality data. In addition, we also empirically evaluate various time series imputation techniques, including classical and state-of-the-art methods for air quality data. In summary, the contribution of our work can be described as follows:

1. We review existing techniques for air quality prediction and missing data imputation.
2. We conduct experiments on various air quality datasets to compare the performance of various time series imputation methods using various measures.
3. We provide analysis and evaluation of the performance of techniques.
4. We provide practitioners practical guidance on how to deal with missing data in air quality data.

The structure of the paper can be organized as follows. Firstly, Section 1 gives an overview of the current research related to data and missing values and describes the research problem in Section 2. Afterward, we review the prediction methods for air quality data in Section 3. Besides, we also review related techniques imputing missing values in time series data from conventional to modern data in Section 4. Next, in Section 5, we present methods for imputing the missing values in this paper. Experiments compare and evaluate the results and imputation time of the methods and the accuracy of prediction models on air pollutant values and AQI levels on the different datasets in Section 6. Then, we discuss the related problems to impute missing values in Section 7. Finally, the paper ends with our conclusion and future works in Section 8.

## 2 Problem formulation

Most urban areas worldwide, including Vietnam, are facing increasing air pollution. Among them, the problem of air pollution due to dust is still the most prominent. In some large cities like Hanoi, the number of days with *PM*10 and *PM*2.5 dust pollution levels exceeding the limits is relatively high. The problem is how to reduce the impact of air pollution on human health. Therefore, to solve the above problem, experts believe that if air pollution is informed early in the form of prediction, it can help people proactively plan their lives, especially on days when air pollution is high, minimizing the effects of air pollution on health. Thereby, people will know and choose how to protect their health and that of their family members. Many countries predict air quality from three to five days in advance based on air and meteorological data (such as temperature, humidity, wind direction, and topography) from air monitoring stations. However, in implementing the problem of collecting data through sensors, the possibility of data loss of information occurs very often and unavoidably. Through this paper, we also present ways to handle missing data and how it will affect the problem of air pollution prediction or similar time series problems.

Missing data can exist in various ways, for example, at individual points or over intervals, where one sensor loses data for a period of time. In this section, we introduce preliminary definitions and formalize the problem of air quality imputation. Air quality data is generally collected from a set of sensors over different periods. We focus on the time series data with missing values. Some notations are defined to describe this problem.

For the rest of the paper, we denote a multivariate time series with *D* variables of time length *T* as $X={\left\{x_{1},x_{2},\ldots ,x_{t},\ldots ,x_{T}\right\}}^{T}\in R^{T\times D}$, where for each *t* ∈ {1, 2, …, *T*}, $x_{t}=\left\{x_{t}^{1},x_{t}^{2},\ldots ,x_{t}^{D}\right\}\in R^{D}$ represents the *t*-th observation vector at time *t* for all variables and $x_{t}^{d}$ denotes the measurement of *d*-th variable of *x*_*t*_, where *d* = 1, …, *D*. Let $s_{t}\in R$ denote the time-stamp when the *t*-th observation is obtained, and we assume that the first observation is made at time-stamp 0. A time series *X* can have missing values. We introduce a masking vector *m*_*t*_ ∈ {0, 1}^*D*^ to denote which variables are missing at time step *t*; the mask token is set to 0 for missing structured data, and the others are set to 1. To represent the missing variables in *X*, the missing mask vector $M\in R^{T\times D}$ is introduced, where

$$\begin{array}{c} M_{t}^{d}=\{\begin{array}{cc} \;1, & \text{if}\;X_{t}^{d}\;\text{is}\;\text{observed}\\ \;0, & \text{otherwise} \end{array} \end{array}$$ (1)

In addition, an indicating mask vector *I* is introduced to differentiate originally missing values and artificially missing values.

$$\begin{array}{c} I_{t}^{d}=\{\begin{array}{ccc} & 1, & \text{if}\;X_{t}^{d}\;\text{is}\;\text{artificially}\;\text{masked}\\ & 0, & \text{otherwise} \end{array} \end{array}$$ (2)

Moreover, the missing ratio for the dataset is defined as follows:

$$\begin{array}{c} p=\frac{\text{Number}\;\text{of}\;\text{the}\;\text{missing}\;\text{values}}{\text{Total}\;\text{number}\;\text{of}\;\text{values}}. \end{array}$$ (3)

For example, we assume that *X* is the input time series matrix with three variables, *M* is the masking matrix for *X*, and *T* is the time stamp for *X*. In the following, we denote $x_{t}^{d}=NaN$ if the value is missing. For example, assume we have nine observations (*T* = 9), (*D* = 3), then these matrix are, respectively,

$$\begin{array}{c} X=(\begin{array}{ccc} 2 & 2 & 1\\ 1 & NaN & 2\\ 3 & 3 & NaN\\ 4 & 6 & 1\\ NaN & 4 & 5\\ NaN & 6 & 3\\ 7 & NaN & 5\\ 8 & 4 & 3\\ 7 & 6 & NaN \end{array}),\;T=(\begin{array}{c} 0\\ 0.1\\ 0.6\\ 1.2\\ 1.6\\ 2.2\\ 2.5\\ 3.1\\ 3.5 \end{array}),\;M=(\begin{array}{ccc} 1 & 1 & 1\\ 1 & 0 & 1\\ 1 & 1 & 0\\ 1 & 1 & 1\\ 0 & 1 & 1\\ 0 & 1 & 1\\ 1 & 0 & 1\\ 1 & 1 & 1\\ 1 & 1 & 0 \end{array}) \end{array}$$ (4)

If this matrix contains any missing values *NaN*, we estimate these values using imputation techniques. We create the masking matrix *M*, each with *NaN* value, which is replaced by 0 and the others by 1.

The performance of each imputation model is computed by considering the indicating mask. The missing values in the matrix *X* will be imputed using traditional imputation techniques (i.e., Mean, Median, MICE, kNNI) and recently developed imputation techniques (i.e., SAITS, BRITS, MRNN, Transformer). In what follows, we will review the current approaches in detail.

## 3 Air quality prediction: Existing techniques

In the current studies, there is a wealth of research on air quality prediction due to its importance in informing about the pollution level that will allow policy-makers to adopt measures for reducing its impact [21, 22]. Methods for air quality prediction can be classified into statistical, machine learning [23, 24], and deep learning approaches.

### 3.1 Statistical methods

#### 3.1.1 Vector Auto-Regression (VAR)

One of the most popular statistical models for forecasting multivariate time series is the Vector Auto-Regression (VAR). It is considered an extension of the univariate autoregressive model. The findings of [25] have revealed that the VAR model is particularly valuable in capturing the dynamic characteristics of economic and financial time series, making it a powerful tool for describing their behavior and making forecasts. In [26], VAR was used to forecast daily concentrations of air pollutants (i.e., *CO*, *NO*_2_, and *SO*_3_) in Tehran city for the next 24h. For such a task, the authors have considered the correlations between air pollutants to get more accurate forecasts. Experimental results have indicated the high efficiency of the proposed method.

#### 3.1.2 Autoregressive Integrated Moving (ARIMA)

Aside from VAR, ARIMA algorithms [27] were applied to forecast air quality. In [28], authors proposed a hybrid method named ARIMAX by combining the advantage of ARIMA and numerical modeling to forecast real-time air pollutants in Hong Kong (i.e., *PM*2.5, *O*_3_, and *NO*_2_). By employing experimental analysis, the proposed method significantly improves the quality of forecast results in multiple evaluation metrics. Similarly, the findings in [29] have shown the prominent role of ARIMA in forecasting PM10 in Dakar, Senegal. Accordingly, the proposed method combines system observations with multi-agent real-time simulation and evaluates with several simulations.

### 3.2 Traditional machine-learning methods

Some traditional machine learning algorithms used for air quality prediction can be Support Vector Regression (SVR), Random Forest (RF), and Linear Regression (LR).

#### 3.2.1 Support Vector Regression (SVR)

SVR models were used to forecast *PM*2.5 and *PM*10 in London [30]. In that paper, the experimental results indicate the SVR’s efficiency in forecasting air quality parameters (i.e., PM2.5 and PM10). A nonlinear dynamic model based on the SVR technique was proposed to forecast AQI in Oviedo, Spain [31]. Accordingly, the proposed model first analyzed the relationship between primary and secondary pollutants. Then, it derived vital factors influencing the air quality and recommended potential enhancements for health and lifestyle. Zhu et al. [32] investigated an application of the SVR algorithm with a quasi-linear kernel for air quality prediction. For such a task, the paper designed a gated linear network to construct the multiple piecewise linear model, and it could be developed through the pre-training of a Winner-Take-All (WTA) autoencoder. This approach could outperform other state-of-the-art methods in the case of complex air quality prediction problems. It is due to the WTA strategy reducing the risk of overfitting and choosing appropriate sparsity parameters.

#### 3.2.2 Random Forest (RF)

Regarding RF [33, 34] proposed a parallel approach combined with Spark to forecast *PM*2.5 in Beijing. The experimental results revealed the efficiency and scalability of the proposed method in the case of big data. Later, RF was used to select the most important features to improve the quality of real-time air quality prediction [35]. Concretely, the proposed method provides highly accurate predictions of three air pollutants (i.e., *PM*2.5, *NO*_2_, *SO*_2_) and outperforms other state-of-the-art methods.

#### 3.2.3 Linear regression

Linear regression is also a state-of-the-art model for air quality prediction. Indeed, many linear regression models have been proposed to predict AQI levels in New Delhi [36]; In Catalonia [37], authors combined factors including the effect of the surface reflectance capacity of urban surfaces with solar radiation and elevation to predict AQI level in Catalonia. The dataset is collected from 75 different air quality monitoring stations. A clustering technique was applied to cluster these stations based on their similarity. Meanwhile, Multiple Linear Regression (MLR) was used to replicate the annual mean values of AQI in Catalonia. Experimental results illustrated that the proposed model provided highly accurate predictions of AQI. Djuric et al. [38] proposed a multiple linear regression to forecast air pollution indices (i.e., *SO*_2_, *NO*_2_, *PM*10, *O*_3_, and *CO*) in Belgrade, Serbia. They collected the training and testing sets from the winters of 2011 and 2012/2013, respectively. In addition, the findings show that the proposed model can be scaled up to forecast long-term air quality.

### 3.3 Deep learning techniques

#### 3.3.1 Long short-term memory

Apart from the traditional machine learning approaches, most deep learning methods, such as Long short-term memory (LSTM), have shown their superiority over many machine learning techniques. Even though in [39], the LSTM model has outperformed MLP and RNN models in predicting *PM*10 and *SO*_2_ in the Basaksehir district of Istanbul province. In [40], authors have proposed a bidirectional LSTM (Bi-LSTM) model by considering both past and future information to forecast PM2.5 of three cities in Korea and five cities in China. Accordingly, its performance is superior to GRU and LSTM in terms of the air quality forecast for these cities. Concretely, with short-term prediction, these models have similar performances. Meanwhile, with long-term prediction, Bi-LSTM outperformed GRU and LSTM. Wang and colleagues [41] developed a combination of the CT (chi-square test) with the LSTM model to analyze the relationship between air pollution variables. The paper identified the factors influencing air quality by using CT for such a task. Then, the AQI level was predicted by the LSTM model using a dataset collected at Shijiazhuang in the Hebei Province of China. In comparison to other competitive methods (i.e., SVR, MLP, BP neural network, Simple RNN), the proposed method provides an accuracy of 93.7% (the highest one). In addition, the proposed method outperforms the baseline methods in terms of MAE, MSE, and RMSE metrics. In [42], a GRU layer has been added to the LSTM structure to improve the accuracy of the air quality prediction problem. Experimental results with a dataset collected in Delhi show the outperformance of the proposed approach compared to other competitive methods of linear regression, GRU, kNN, and SVM in terms of MAE and *R*^2^.

#### 3.3.2 Recurrent Neural Networks (RNN)

In [43], the authors have proposed an RNN algorithm to predict PM2.5 in Japan by employing a dynamic method to pre-train the model based on multi-step-ahead time series prediction. [44] apply RNN to predict *PM*10, *O*_3_, *SO*_2_, *CO* and *NO*_2_. The dataset is collected from different sensors with intervals of 1 hour. In addition, the authors applied fine-tuning to find the best hyperparameters of neural network structure and optimization function. Moreover, the investigated model can be applied to predict similar pollutants in other neighboring areas.

#### 3.3.3 Gated Recurrent Unit (GRU)

In the current literature, many research results indicate that existing models are best at short-term forecasts. Meanwhile, improving existing approaches to forecasting long-term air quality is necessary. [45] proposed an algorithm that is considered an enhanced version of GRU (named BiAGRU) by combining bidirectional gated recurrent unit integrated with an attention mechanism. By means of experimental analysis, the proposed model is superior to many traditional machine learning models and modern deep learning models.

Referring to [46], a model based on Gated Recurrent Units (GRUs) has been proposed to forecast *NO*_2_ pollutant concentration. The proposed model is assessed and fine-tuned for such a task concerning the number of features, look-backs, neurons, and epochs. Also, in Beijing [47], authors introduced a model based on spatiotemporal CRUs combined with a Geographic Self-Organizing Map (GeoSOM). Concretely, all monitor stations were clustered using time-series features and geographical coordinates. Later, GRU models were proposed for clusters, and Gaussian vector weights were used to weigh different models in predicting the target sequence. Experimental results showed the technique’s efficiency compared to several state-of-the-art ones regarding MAE, MRE, and R2 metrics.

Since existing models do not fully consider the temporal dependencies, spatial correlations, and feature correlations hidden in a given dataset, in [48], authors examined these correlations by introducing a spatiotemporal deep learning model named Conv1D-LSTM based on 1-D convolutional neural network and LSTM for spatial and temporal correlation feature extraction. In addition, a fully connected network exploits these features for the air quality prediction problem. Furthermore, missing data have been imputed to enhance the quality of air quality prediction. The proposed method outperformed other well-known baseline methods through experimental analysis.

### 3.4 Data fusion

Besides techniques for the air quality prediction problems as mentioned above, there exist further works solving the problem by using data fusion. In this section, we will provide a brief discussion of these approaches.

#### 3.4.1 Multimodal data

Air pollution is one of the most worrying issues facing the world today. So, forecasting of particulate matter (PM) is necessary nowadays. Ton et al. [49] pointed out that combining meteorological features and timestamp information in Hanoi air quality datasets improved the results of PM10 and PM2.5 forecasting. The authors extracted two new features, which were *weekend* and *working hour*, from the “Date Time” recorded variable. Then, encoding the time into a vector of 0, 1 to include two new variables, *weekend and working hour*. First, with the variable *weekend*, the time vector from Monday to Friday was encoded as 0. On the other hand, during Saturday and Sunday weekends, the time vector was 1. Second, with *working hour*, the time vector was 1 in the range 7 AM to 7 PM, whereas this was 0. According to the authors, the time steps of the two new variables *weekend and working hour* were synchronized with other weather and air quality variables. It was highly efficient in 68% of the cases compared to other methods by conducting five deep learning models: MLP, 1D-CNN, LSTM, Bi-LSTM, and Stacked LSTM. Besides, in the long-term forecast of PM concentrations, the Vanilla LSTM model with combined features performed better than the other.

Similarly, to predict the *PM*2.5 air pollution level in the short- and medium-term, Tejima and colleagues [50] also proposed a framework that looks for hidden associations between traffic factors and air pollution. The six steps in their framework can be defined as follows: (1) Use any machine learning algorithm to extract features from the traffic images, (2) Create a new dataset by combining the extracted features dataset and air pollution dataset using time, (3) Use fuzzy rules to convert this new dataset into an uncertain temporal database, and (4) Use uncertain periodic-frequent pattern mining techniques to uncover hidden relationships between various traffic factors and air pollution, (5) estimate air pollution level from a given image using transfer learning on a pre-trained model, and (6) predict air pollution level using estimated air pollution level and mined patterns dataset. Experimental results show that their method can accurately estimate and predict air pollution levels, ranging from 77% to 98%.

#### 3.4.2 Neighbor stations

Currently, air pollution and urban life influence human health. Therefore, environmental and data science experts always try to find the most accurate way to predict and provide timely warnings to humans. Specifically, Dao et al. [51] use methods of data imputation for the UrbanAir dataset to predict air pollution at a place without a station by using neighbor stations and predict air pollution of Dalat and discover the correlation/association between air pollution and human activities. The authors divided the article into two tasks that need to be performed: Subtask 1 only used environmental data to predict air pollution and only used traffic data in Subtask 2. Subtask 2 accepts training a prediction model using environmental and traffic data, but only traffic data is used to predict air pollution. While Subtask 2 only accepts AQI levels, Subtask 1 requires predicting both the exact value and AQI level of each pollutant concentration. The paper encouraged researchers to develop a generic framework to discover a correlation among different traffic factors, weather, and air pollution in a locality. By using these correlations, the authors improved the accuracy of AQI prediction and understood the mutual impact between urban life and air pollution.

Besides, Nguyen et al. [52] also introduced a dataset containing data about personal life and the surrounding environment, collected periodically along predetermined routes in Ho Chi Minh City, Vietnam. They also introduced self-developed devices and system architectures for data collection, storage, access, and visualization. There were interesting research topics and applications, including understanding the correlation between human health, air pollution, and traffic congestion.

#### 3.4.3 Images

Human health is mostly impacted by air pollution. Over time, there has been an increase in the number of patients and disease reports related to air pollution. By using lifelog data and urban nature similarity, a method was introduced in [53, 54] that could predict AQI at a local and individual scale with a few images taken from smartphones and open AQI and weather datasets. Various public datasets pertaining to weather, air pollution, and images are used to develop and evaluate image retrieval and prediction model techniques. The outcomes support their hypothesis regarding the strong correlation between the AQI and snapshots of the surrounding area.

#### 3.4.4 Variable selection

Currently, several statistical and machine learning methods are used to uncover useful information and patterns for enormous datasets. The common model selection (variable selection) methods include Neural Networks (NN) and RF. The statistical methods like the Least Absolute Shrinkage And Selection Operator (LASSO) [55] and principal component analysis (PCA). The authors [56] have proposed combining NN with LASSO or RF for even better results. In addition, they tested these new methods along with classical techniques (ordinary least square and feed-forward NN) using Monte Carlo simulation and real-world air quality data from Italy. The study found that the combined methods achieved lower errors, suggesting they outperform the traditional approaches.

Many methods have been proposed to improve the performance of air quality prediction. However, most investigated methods are based on complete datasets. Therefore, we need to impute missing values to reinforce the prediction models’ performance.

## 4 Data imputation: Recent techniques

Various statistical and machine learning methods [57–59] have been developed to overcome the problem of missing data for time series, to fill in the missing values in the data, or in other words, imputing the missing values. However, methods have limitations in handling data with high missing rates or changes in available variables. In addition, the performances of these methods vary widely according to the type of data, noise levels, or other factors and show a high dependence on correlations within the data.

In this section, we want to provide an overview of the relationships among the given imputation techniques and comparisons and then discuss them individually.

### 4.1 Conventional methods

#### 4.1.1 Ignoring

Ignoring [60, 61] is a method that completely ignores missing values when conducting the analysis process. Although this is a simple method, if the rate of missing data is high enough to influence the analysis outcomes, it is highly dangerous.

#### 4.1.2 Deletion

An approach of removing/deleting missing observations from raw data is called Deletion [62, 63]. It is also a frequently used method when the missing values of the data are not high, and removing missing values will not affect the analysis results. Nevertheless, when the missing data rate is high, deleting missing values makes the data incomplete and unsuitable for some other analysis applications.

#### 4.1.3 Mean/Median/Mode imputation

Mean/Median/Mode are simple methods. There, the missing value for a continuous variable is imputed by the mean/median of the observed values. When the missing values for a categorical variable are replaced by the Mode of the observed values, these approaches are quick to compute and simple to implement. Mean/Median/Mode Imputation methods [64] are a solution for better analysis results when they solve the issue of handling missing data values, whereas Ignoring and Deletion methods are thought to provide poor results in the analysis or data mining process when the missing data rate is high. Furthermore, the limitation of these methods is that the bias created by multiple values on the data has the same value, even if the data are MCAR. As a result, it may bias the estimation of skewed distributions.

#### 4.1.4 Regression imputation

There are two steps in Regression imputation [65, 66]. The first is to estimate a linear regression model using the target variable’s observed values along with the explanatory variables. After that, one can use the model to predict values for the missing cases in the target variable. Missing values of the variable are replaced based on these predictions. There are two types. First is deterministic regression imputation. It means missing values are replaced with the exact prediction of the regression model. The second is stochastic regression imputation, which adds an additional random error term to the predicted value imputed by deterministic regression imputation. Regression imputation is the improvement over Mean/Median/Mode imputation. Besides, it has disadvantages, including the assumptions of error distribution and linear relationship, which are relatively strict and give poor results for heteroscedastic data.

#### 4.1.5 Last Observation Carried Forward (LOCF)

Last Observation Carried Forward [67, 68] fills in missing values by using the last observed value of the given features in each sample; if there is no previous observation, 0 will be filled in. LOCF assumes that the missing data is constant or follows a gradual change. However, if the missing values are not stationary or the sensor readings exhibit abrupt changes, this method may introduce bias and inaccuracies.

#### 4.1.6 Multivariate Imputation by Chained Equations (MICE)

Multiple imputation offers numerous benefits compared to the single imputation methods mentioned above. MICE [69, 70] is one of the most popular multiple imputation techniques. The process uses an iterative set of regression models to impute missing data from a dataset. It imputes missing values in the dataset’s variables by focusing on one variable at a time. Once the focus is placed on one variable, MICE uses all the other variables in the dataset to predict missingness in that variable. The prediction is based on a regression model, with the form of the model depending on the nature of the focus variable. MICE methods perform better and are more reliable for data with a limited sample size.

On the other hand, MICE has several benefits, such as results in unbiased estimates, being easily interpreted in a Bayesian context, and having a large number of workable algorithms built into the MICE framework. It is worth noting that MICE is especially helpful when missing values are associated with the target variable in a way that causes leakage. Users can also state what they believe to be the likely distribution of the missing value using MICE. However, MICE comes at a high computational cost.

#### 4.1.7 First five last three logistic regression imputation (FTLRI)

Chen et al. [71] proposed an interesting approach for data imputation, namely FTLRI, for time-series air quality data. The paper is based on the traditional logistic regression and a presented *“first Five & last Three”* model. These techniques could explain relationships among disparate attributes and then derive highly relevant data, for both time and attributes, to the missing data, respectively. The results showed that FTLRI has a significant advantage over the compared imputation approaches, particularly in short-term and long-term time-series air quality data. Furthermore, FTLRI can perform better on datasets with relatively high missing rates (about 40%) since it only selects highly relevant data to the missing values instead of relying on all other data like other methods.

#### 4.1.8 Autoregressive Distributed Lag (ARDL)

Selecting criteria is considered an important issue in the Autoregressive Distributed Lag (ARDL) model. El et al. [72] proposed the use of four imputation methods (k-Nearest Neighbors, Expectation-Maximization, Classification, and Regression Tree, and Random Forest) for handling the missing values. Their goal was to improve the accuracy of the model with the optimal order of lags. They compared these methods using real economic data related to foreign direct investment (FDI) in Libya. Their findings suggest that the Expectation-Maximization method performed best compared to the others.

#### 4.1.9 EPK

Next, Mohamed et al. [73] introduced a new imputation technique called EPK. Using the Monte Carlo simulation, they evaluated the effectiveness of nine different imputation methods, including EPK. The simulations focused on a specific type of statistical model (binary logistic regression) when the missingness mechanism is MAR. Additionally, they tested the methods on real data from social network advertising. The results from both simulations and real-world applications showed that EPK outperformed other imputation methods regardless of where the missing data occurred (independent variables only, dependent variable only, or both).

### 4.2 Machine-learning approaches

The recent methods for imputing missing data in time series led to more accurate and improved imputed data than traditional approaches. Choosing an appropriate imputation method for a specific type of missing data significantly impacts the performance of data imputation.

#### 4.2.1 k-Nearest Neighbor Imputation (kNNI)

kNNI method [14, 69] uses the k-nearest neighbor to identify similar samples with normalized Euclidean distances or some other type of distance and impute the missing values with the average value of its neighbors. The k-nearest neighbor method can impute continuous variables (by using the mean or weighted mean among the k-nearest neighbors) and categorical variables (by using the Mode among the k-nearest neighbors). Both quantitative and qualitative features are handled by kNNI with ease. However, it performs computationally intensively for large data since it searches through all the datasets and requires the specification of hyper-parameters that can greatly affect the results.

#### 4.2.2 MissForest

The Random Forest (RF) algorithm can also applied for multivariate time series data, employing an average of the corresponding full values. Using proximity data points, this algorithm then iteratively improves the imputation of missing data. Generally, missForest is a technique that was proposed by [74] based on Random Forests. The article showed that RF intrinsically constitutes a multiple imputation scheme by averaging many unpruned classification or regression trees. The imputation error can be estimated without a test set using Random Forest’s built-in out-of-bag error estimates. Furthermore, missForest performs better than K-nearest neighbors and other imputation techniques, giving outstanding results for data containing non-linear relations and/or complex interactions. Additionally, it works well with data containing both qualitative and quantitative features. When using missForest, there is no need to tune parameters, do categorical encoding, or standardize the data. MissForest can be utilized to achieve good imputation results even in high-dimensional datasets with a large number of variables compared to the sample size.

### 4.3 Deep neural networks

In addition, many deep learning techniques have been developed to solve imputation for missing values in time series data.

#### 4.3.1 GRU-D

Chen et al. [75] proposed the GRU-D model, which is a deep learning model based on Gated Recurrent Unit (GRU) that takes two representations of missing patterns, i.e., masking and time interval, and effectively incorporates them into a deep model architecture so that it does not only captures the long-term temporal dependencies in time series but also utilizes the missing patterns to achieve better prediction results.

#### 4.3.2 Deep auto-encoder

One method that can be used for data imputation is the auto-encoder structure. It extracts features from low-dimensional layers using the encoder and decoder structure, and the decoder recovers missing values. As such, it can function as a methodological feature. [76] presented one technique using deep autoencoders for spatiotemporal challenges involving imputing missing data. The proposed method for capturing temporal and spatial patterns was a convolution bidirectional LSTM. Additionally, the authors analyzed an autoencoder’s latent feature representation in spatiotemporal data and illustrated its performance for missing data imputation. The experimental result illustrated that the convolution recurrent neural network outperforms state-of-the-art methods.

#### 4.3.3 MultiLayer Perceptron (MLP)

Next, [77] estimated the missing values of a variable in multivariate time series data using a MultiLayer Perceptron. To achieve the best prediction performance for the specified time series, an automated technique was employed to identify the optimal MLP model architecture, filling in a long continuous gap instead of relying on isolated, randomly missing observations. The findings demonstrated that using MLP to fill a big gap produces better outcomes, especially when the data behaves nonlinearly.

#### 4.3.4 Raindrop

Raindrop [78] is a Graph Neural Network-based algorithm embedding irregularly sampled and multivariate time series. It is inspired by how raindrops hit a surface at varying time intervals and create ripple effects propagating throughout the surface. Raindrop helps handle missing data with irregular time series. It represents every sample as a separate sensor graph and models time-varying dependencies between sensors with a novel message-passing operator. It estimates the latent sensor graph structure and leverages the structure together with nearby observations to predict misaligned readouts. This model can be interpreted as a graph neural network that sends messages over graphs that are optimized for capturing time-varying dependencies among sensors. Another typical work comes from Festag et al. [79], where the authors developed a system based on Generative Adversarial Networks that consist of recurrent encoders and decoders with attention mechanisms and can learn the distribution of intervals from multivariate time series conditioned on the periods before and, if available, periods after the values that are to be predicted.

Therefore, it is worthwhile to understand the data types with missing values and propose an effective and robust strategy to fill time-series air quality data with missing values.

## 5 Data imputation for air quality prediction

**Fig 1:**
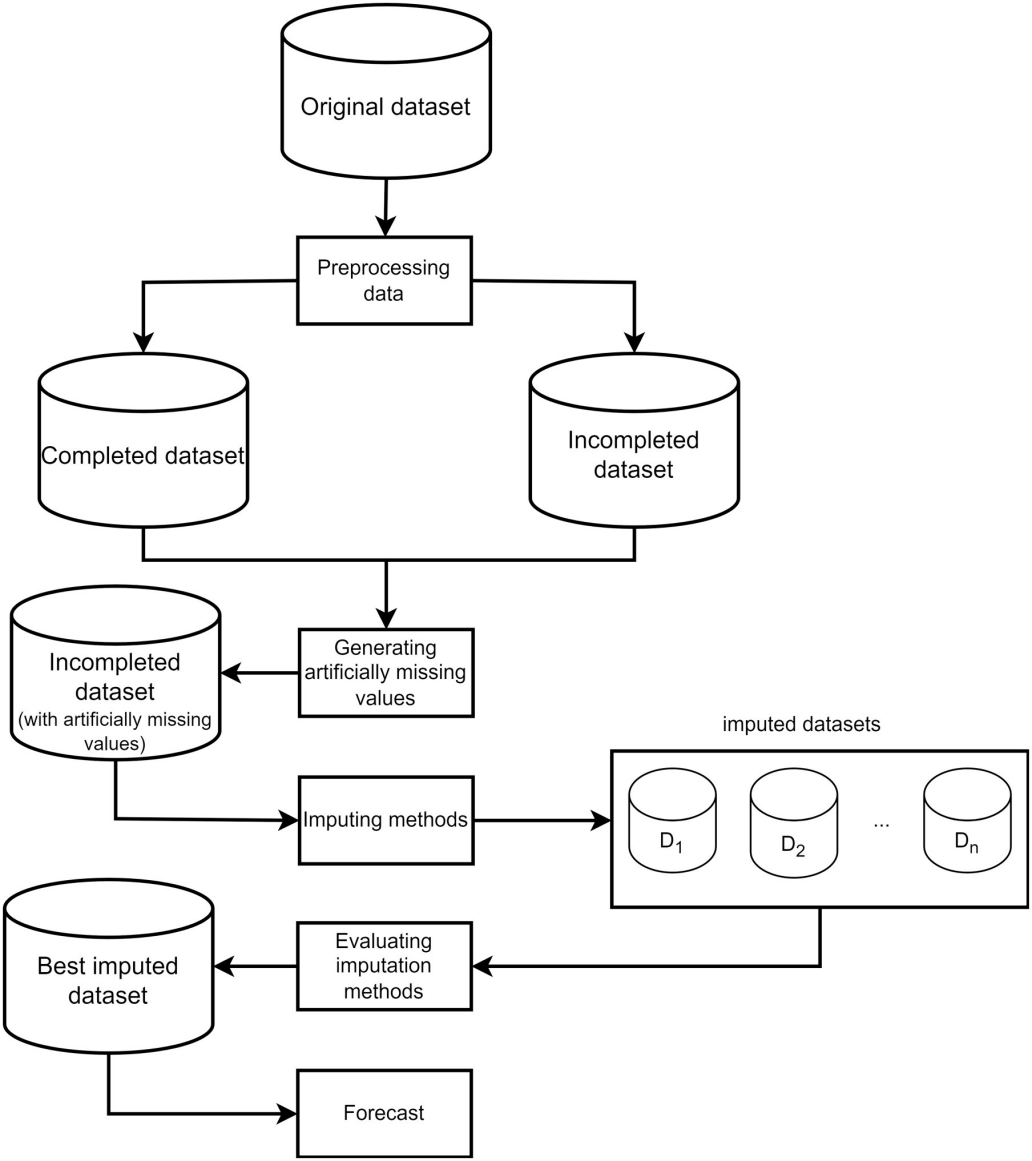
A normal setup of a training process for Machine Learning and Deep Learning frameworks.

A flowchart for the setup of the training process for the Machine Learning and Deep Learning framework proposed in this work is shown in Fig 1.

In experiments, to obtain a thorough comparison, we compare some classical by widening traditional imputation methods with some recently developed imputation methods:

- **Mean Imputation** [80]: The missing values are replaced with the mean value of the corresponding features.
- **Median Imputation** [81]: It is similar to Mean imputation, but the median is utilized instead of the mean.
- **Multivariate Imputation by Chained Equations (MICE)** [69]: MICE imputes missing values in the variables of the dataset by focusing on one variable at a time. Once the focus is placed on one variable, MICE uses all the other variables in the dataset to predict missingness in that variable. The prediction is based on a regression model, with the form of the model depending on the nature of the focus variable.
- **k-Nearest Neighbor Imputation (kNNI)** [69]: It uses the k-nearest neighbor method to identify similar samples and impute the missing values with the average value of its neighbors. The k-nearest neighbor method can impute continuous variables (the mean or weighted mean among the k-nearest neighbors) and categorical variables (the mode among the k-nearest neighbors).

The main deep learning methods researched for time series imputation are SAITS [13], BRITS [12], MRNN [82], and Tranformer [83, 84]. All of them are deep learning approaches published recently for time series imputation.

- **Self-attention-based imputation for time series (SAITS)** [13]: a self-attention mechanism for missing value imputation in multivariate time series. Typically, it is trained by a joint-optimization approach. SAITS learns missing values from a weighted combination of two diagonally masked self-attention (DMSA) blocks. DMSA explicitly captures both the temporal dependencies and feature correlations between time steps, which improves imputation accuracy and training speed. Meanwhile, the weighted-combination design enables SAITS to dynamically assign weights to the learned representations from two DMSA blocks according to the attention map and the missingness information.
- **Bidirectional Recurrent Imputation for Time Series (BRITS)** [12]: a method for filling the missing values for multiple correlated time series. It learns the missing values in a bidirectional recurrent dynamical system without any specific assumption. The imputed values are treated as variables of the RNN graph and can be effectively updated during the backpropagation.
- **Multi-directional Recurrent Neural Network (MRNN)** [82]: is a neural network architecture including two blocks (interpolation and imputation) trained simultaneously. The interpolation process operates within data streams, while the imputation process operates across data streams. The interpolater uses a Bi-directional Recurrent Neural Network (Bi-RNN) to interpolate missing values within each channel along the time dimension. Afterward, using a simple, fully connected neural network, the imputer can compute an estimate for each time step along all channels.
- **Transformer** [83, 84]: Transformer is a self-attention-based model. It uses transformer architecture in an unsupervised manner to perform missing value imputation. Unlike the existing transformer architectures, this model only uses the encoder part of the transformer due to computational benefits. It is a joint-optimization training approach of imputation and reconstruction for self-attention models to perform missing value imputation for multivariate time series.

In the following sections, we will compare different data imputation techniques with various datasets related to air quality prediction.

## 6 Results

### 6.1 Experimental setup

The efficacy of the missing data imputation methods depends heavily on the problem domain, for example, sample size, types of variables, and missingness mechanisms.

We evaluated the methods mentioned in Section 5 on six real datasets. These datasets include cases with small, moderate, and large sample sizes: Air quality in Frankfurt, Germany (Available on: https://www.kaggle.com/datasets/avibagul80/air-quality-dataset); Beijing Multi-Site Air Quality (Available on: https://archive.ics.uci.edu/dataset/501/beijing+multi+site+air+quality+data) [13, 85]; Air quality in Northern Taiwan (Available on: https://www.kaggle.com/datasets/nelsonchu/air-quality-in-northern-taiwan); Air Quality in Dalat, Vietnam (Available on:https://github.com/BinhMisfit/air-pollution-datasets/tree/main/Dalat-air-quality-dataset) [51] and Air quality dataset in Minh Khai district and Cau Giay district in Hanoi, Vietnam (Available on: https://github.com/BinhMisfit/air-pollution-datasets/tree/main/Hanoi-air-quality-dataset) [49].

The descriptions of the six datasets used in this work and their preprocessing details are elaborated on below:

The first dataset is a time-series air quality dataset with categorical contextual information (time and weather); the air pollution PM2.5 values were collected from sense-boxes installed in Frankfurt, Germany. The dataset has been read from 14 different sensors in close spatial proximity. The dataset was efficiently labeled and can be used as a gold-standard dataset for unsupervised problems. Similarly, the test set of this data takes data from 20% original dataset, 20% of the remaining 80% of the original dataset is used for validation, and the remaining is for training. We also chose every 30 minutes of data and every 1-hour consecutive step to generate time series data samples.

The second dataset is Beijing Multi-Site Air-Quality. It includes hourly air pollutant data from 12 monitoring sites in Beijing. This dataset collected data from 01/03/2013 to 28/02/2017 (48 months in total). For each monitoring site, 12 continuous time series variables are measured (*e*.*g*., *PM*2.5, *PM*10, *SO*2). The test set of the third dataset takes data from 20% original dataset, 20% of the remaining 80% of the original dataset is used for validation, and the remaining is for training. The validation set contains data from the following 05/11/2016. The training set takes from 18/12/2015. In addition, we take every one-hour data to generate a time series of data samples for every 24 consecutive steps.

The third dataset is from the Environmental Protection Administration, Executive Yuan, R.O.C. (Taiwan). It was only collected in Northern Taiwan in 2015, containing air quality and meteorological monitoring data. Besides, this data included 25 air pollution stations and 21 features. The test set takes data from 20% original dataset, 20% of the remaining 80% of the original dataset is used for validation, and the remaining is for training. Specifically, the training set takes place on 15/01/2015, the validation set takes place on 01/09/2015, and the remaining part is used as a test set. We selected every 1-hour data and every 12 consecutive steps in experiments to generate time series data samples.

The fourth dataset is Dalat Air Quality. Urban Air provides a streaming dataset from CCTV and air station networks installed in Dalat City, Vietnam. The system runs 24 × 7 and has several real problems, such as sudden camera/station turn-on/switch-off, noise, and outliers. There are ten air pollution stations (i.e., sensors 01-10), three attached to weather stations (i.e., sensor01, sensor02, sensor03), and fourteen CCTV cameras. Furthermore, the test set of the dataset takes data from 20% original dataset, 20% of the remaining 80% of the original dataset is used for validation, and the remaining is for training. We generate time series data samples by selecting every 1-hour data and every 24 consecutive steps.

The two final datasets were collected hourly at two monitoring stations in Hanoi: Cau Giay district and Minh Khai district. For example, Cau Giay dataset with observation time from 25/2/2019 to 25/11/2020, and Minh Khai dataset from 01/1/2019 to 25/11/2020 record measured features including *PM*10, *PM*2.5, *SO*_2_, *O*_3_, *NO*_2_, *NO*, *NO*_*x*_, *CO*, Temperature, Humidity, Wind speed, Rain, Wind direction, Atmospheric Pressure, Solar Radiation. Besides, 20% of the original dataset is utilized for the test set, 20% of the remaining 80% is used for validation, and the remaining is used for training. Then, we choose one hour’s worth of data per 24 consecutive steps to create time series data samples.

After the preprocessing step, general information about the datasets is described in Table 1.

**Table 1:** The summary of six existing datasets for the air quality prediction problems.

Dataset	Features	Samples	Original missing rate
Frankfurt (German)	14	1.230.693	0%
Beijing (China)	19	420.768	0.93%
Northern Taiwan (Taiwan)	23	218.640	23.73%
Dalat (Vietnam)	14	35.280	41.68%
Cau Giay District (Hanoi, Vietnam)	17	14.681	0.49%
Minh Khai District (Hanoi, Vietnam)	17	15.917	2.49%

We generate artificial missingness to evaluate all imputation methods used. It is important to note that normalization is applied in the preprocessing of all datasets. For each dataset, the missing ratio *p* is varied from 10% to 80% with increments of 10% for each dataset to evaluate the models at different missing ratios. For *p* ∈ {10%, 20%, 30%, 40%, 50%, 60%, 70%, 80%}, we train the model to fill the missing values and then calculate the imputation accuracy. However, with the high missing rates in the original Dalat dataset (greater than 40%) for this dataset, we generate extra artificial missing values with missing rates of 10%–30% only.

Besides, the specific information about the architectures of SAITS, BRITS, MRNN, and Transformer models in this paper can be described in Table 2.

**Table 2:** The architectures of SAITS, BRITS, MRNN, and transformer models.

Model	n_layers	d_model	d_inner	n_heads	d_k	d_v	dropout	attn_dropout	Batchsize	rnn_hidden_size	epochs	ORT_weight	MIT_weight	patience	Optimizer
SAITS	2	256	128	4	64	64	0.1	0.1	32	-	15	1	1	3	Adam
BRITS	-	-	-	-	-	-	-	-	32	128	15	-	-	3	Adam
MRNN	-	-	-	-	-	-	-	-	32	128	15	-	-	3	Adam
Transformer	6	512	256	4	128	128	0.1	0	32	-	15	1	1	3	Adam

After the model imputes all missing values, two metrics are used to measure the imputation methods’ performance: Mean Absolute Error (MAE)

$$\begin{array}{c} MAE=\frac{\sum_{d=1}^{D}\;\sum_{t=1}^{T}\;\left|X_{t}^{d,real}-X_{t}^{d,imputed}\right|}{n}, \end{array}$$ (5)

and Root Mean Square Error (RMSE)

$$\begin{array}{c} RMSE=\sqrt{\frac{\sum_{d=1}^{D}\;\sum_{t=1}^{T}\;{\left(X_{t}^{d,real}-X_{t}^{d,imputed}\right)}^{2}}{n}}, \end{array}$$ (6)

where $X_{t}^{d,real}$ and $X_{t}^{d,imputed}$ are real and imputation values, respectively, and *n* denotes the size of dataset with missing values.

For multiple imputation cases with *K* imputations, we have *K* values for MAE and RMSE per dataset, and we use the average to evaluate the model performance. We designed each experiment 10 times. We report mean MAE and RMSE, along with their running time, as the performance metrics.

In this paper, all the models were trained/tested on a computer with the following configurations: Intel(R) Xeon,(R) Gold 6254 CPU @3.10GHz/512GB RAM.

**Fig 2:**
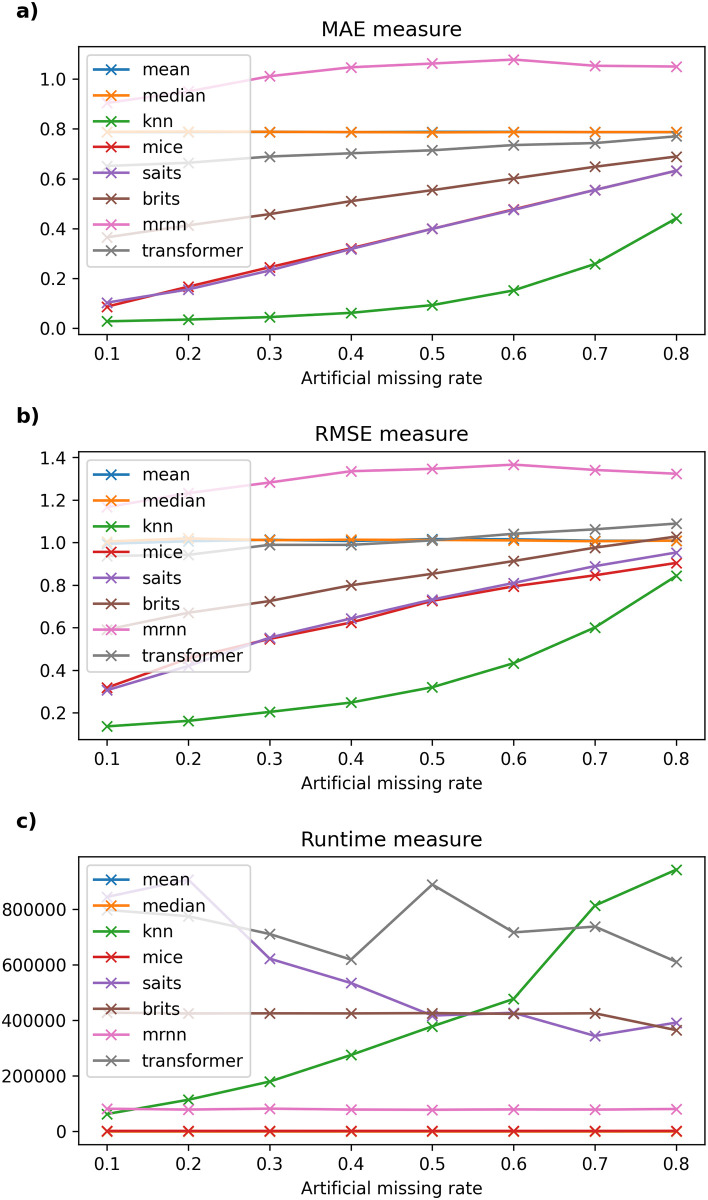
The experimental results on Frankfurt dataset.

**Fig 3:**
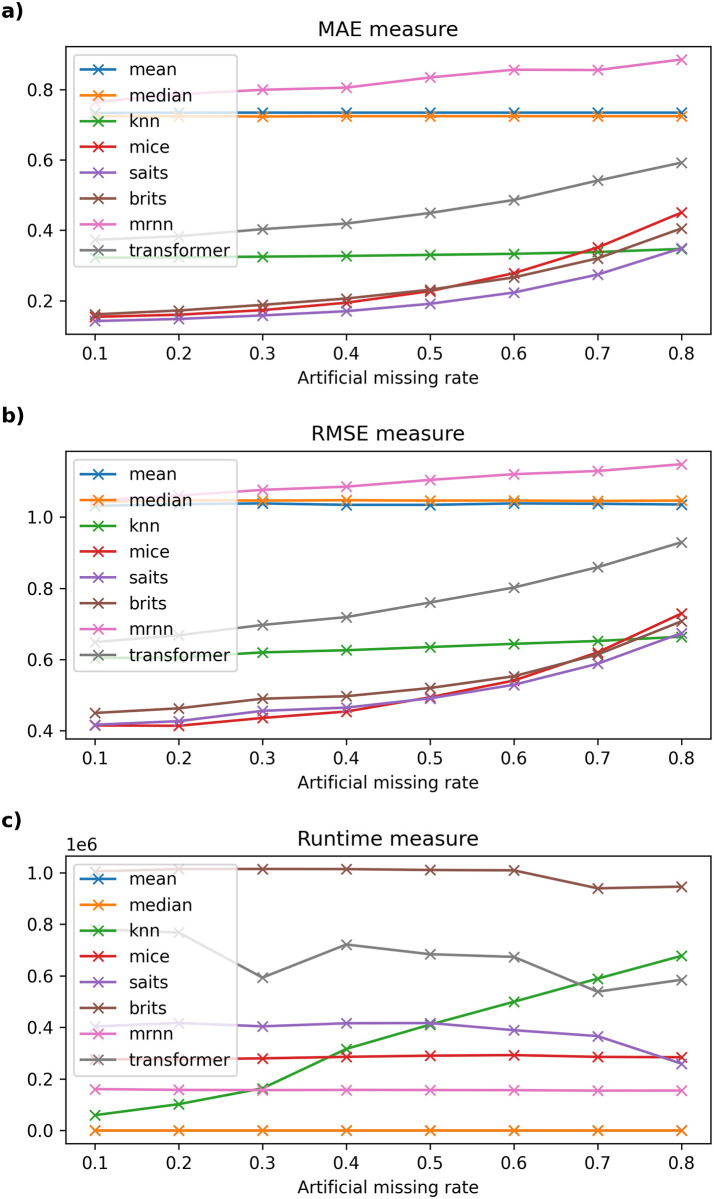
The experimental results on Beijing dataset.

**Fig 4:**
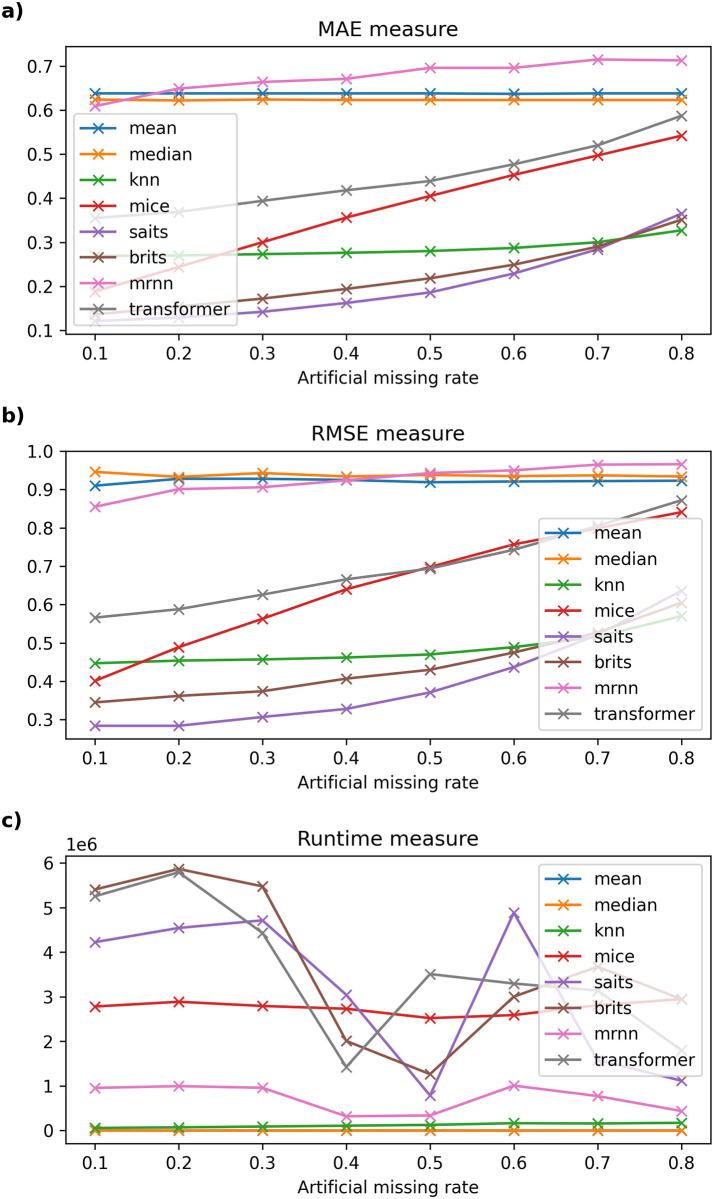
The experimental results on Northern Taiwan dataset.

**Fig 5:**
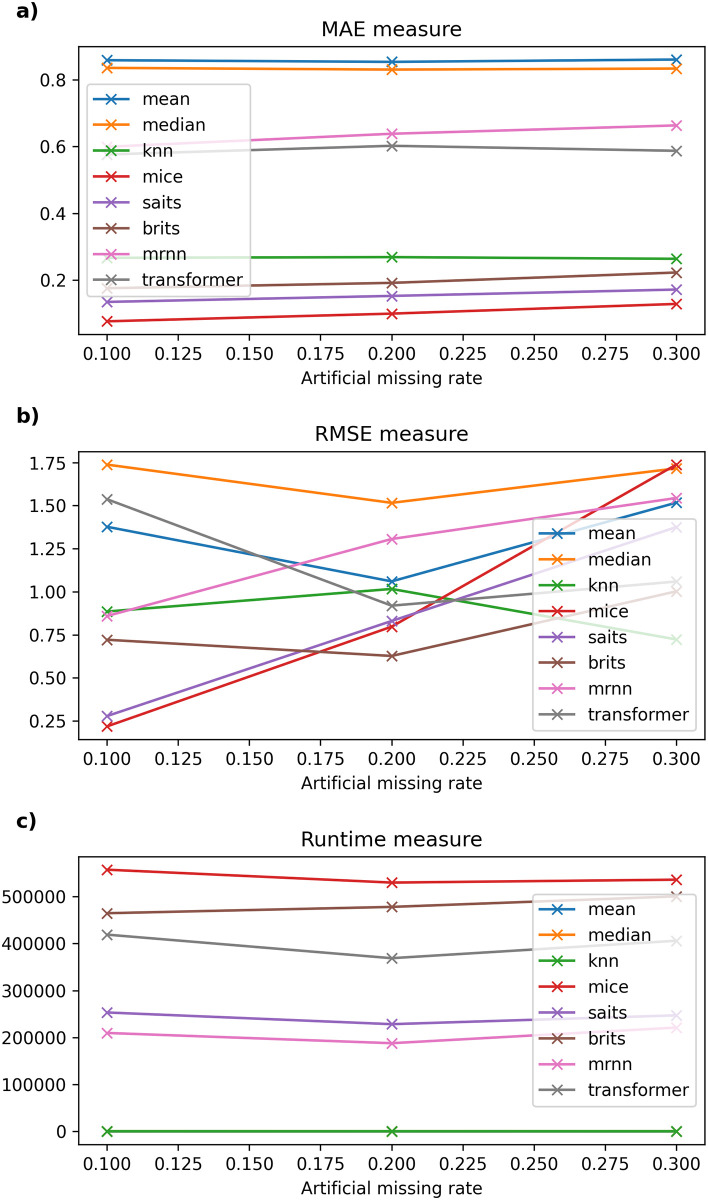
The experimental results in Dalat dataset. Due to the high missing rates in the original Dalat dataset greater than 40%, we generate extra artificial missing values with missing rates of 10%–30% only.

**Fig 6:**
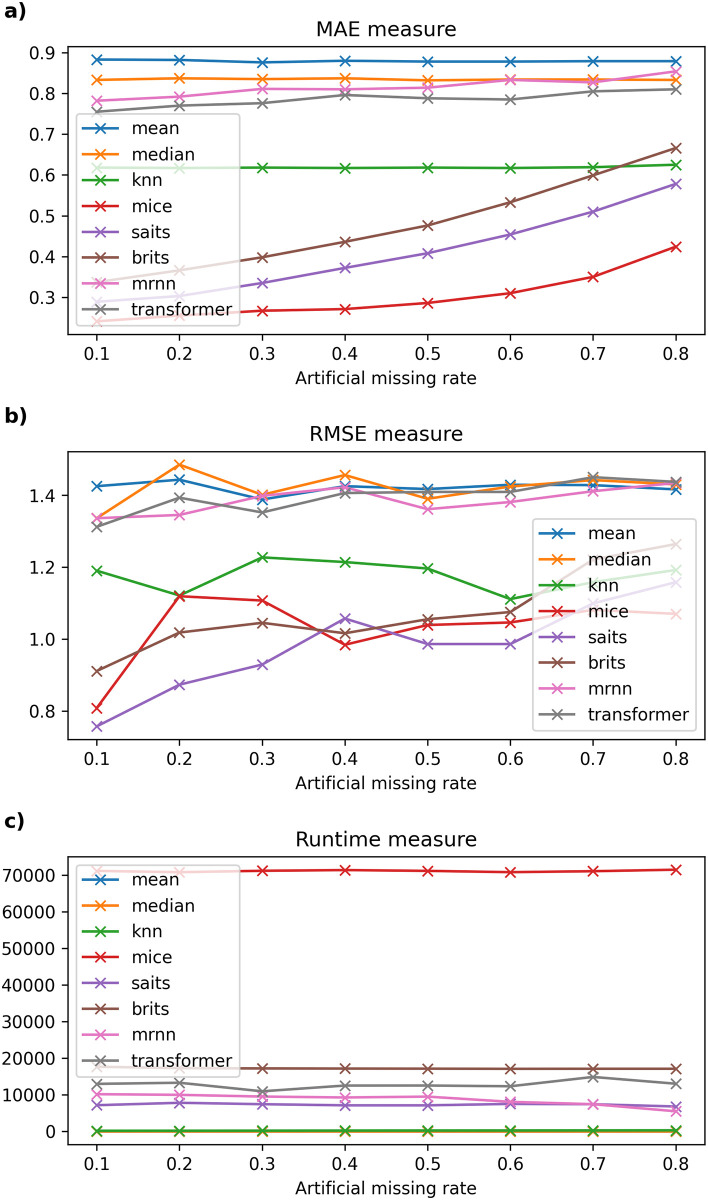
The experimental results in the Cau Giay District dataset.

**Fig 7:**
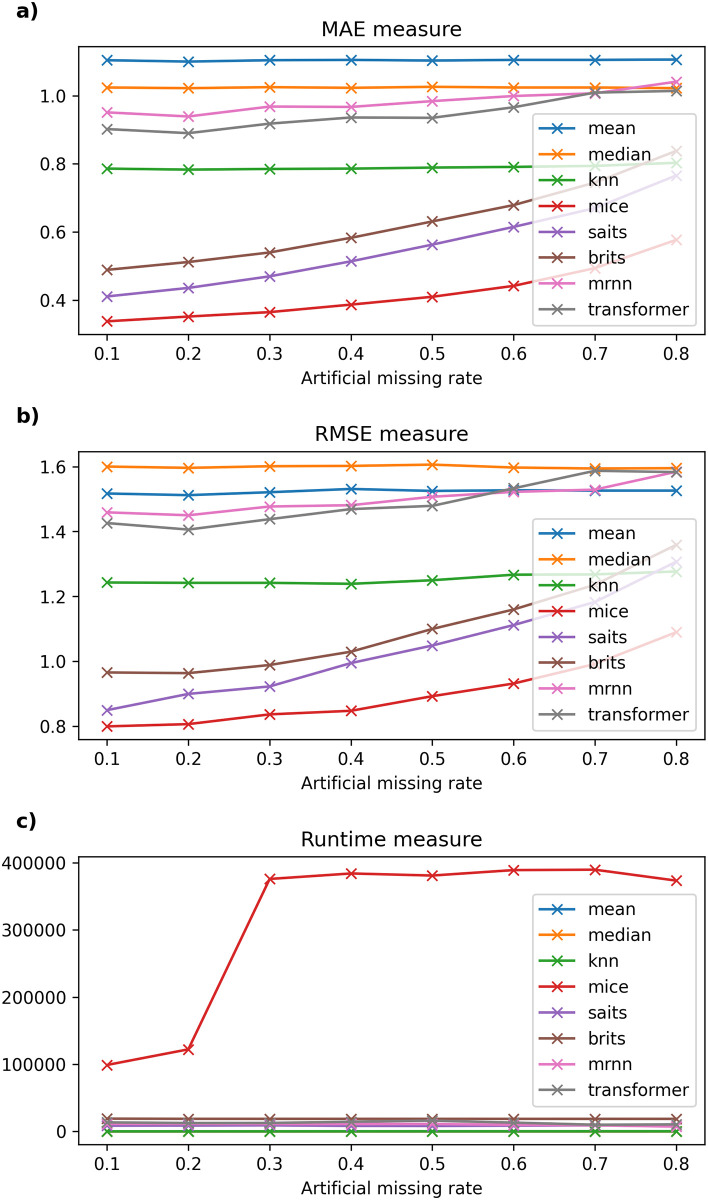
The experimental results on Minh Khai District dataset.

Detailed experimental results and the running time of imputation methods on six datasets are recorded in Tables 3–8 and described in Figs 2–7.

**Table 3:** Experimental results of different methods in Frankfurt air quality datasets.

Missing rate	Measures	Mean	Median	kNNI	MICE	SAITS	BRITS	MRNN	Transformer
10%	MAE	0.787	0.788	**0.028**	0.087	0.103	0.365	0.904	0.651
	RMSE	0.994	1.005	**0.136**	0.318	0.306	0.591	1.166	0.937
	Running time	2.478	5.113	62.784 × 10^3^	1.966 × 10^3^	843.803 × 10^3^	427.553 × 10^3^	81.789 × 10^3^	797.147 × 10^3^
20%	MAE	0.787	0.789	**0.035**	0.167	0.156	0.413	0.95	0.664
	RMSE	1.006	1.019	**0.162**	0.455	0.42	0.67	1.232	0.942
	Running time	3.408	4.792	114.092 × 10^3^	2.030 × 10^3^	906.243 × 10^3^	424.552 × 10^3^	78.815 × 10^3^	774.243 × 10^3^
30%	MAE	0.788	0.788	**0.045**	0.245	0.232	0.458	1.011	0.689
	RMSE	1.013	1.011	**0.204**	0.547	0.553	0.725	1.282	0.989
	Running time	3.931	5.328	179.577 × 10^3^	1.956 × 10^3^	621.778 × 10^3^	425.011 × 10^3^	82.128 × 10^3^	710.461 × 10^3^
40%	MAE	0.787	0.787	**0.062**	0.321	0.318	0.51	1.047	0.702
	RMSE	1.007	1.013	**0.248**	0.624	0.643	0.799	1.335	0.989
	Running time	4.598	5.399	275.131 × 10^3^	1.991 × 10^3^	534.024 × 10^3^	424.715 × 10^3^	78.761 × 10^3^	618.269 × 10^3^
50%	MAE	0.788	0.786	**0.093**	0.399	0.399	0.554	1.062	0.714
	RMSE	1.016	1.012	**0.32**	0.726	0.732	0.853	1.346	1.01
	Running time	4.586	5.504	378.159 × 10^3^	1.990 × 10^3^	416.992 × 10^3^	425.785 × 10^3^	78.066 × 10^3^	888.465 × 10^3^
60%	MAE	0.788	0.787	**0.152**	0.477	0.475	0.601	1.078	0.735
	RMSE	1.015	1.01	**0.433**	0.794	0.81	0.913	1.366	1.041
	Running time	4.614	5.254	476.814 × 10^3^	1.976 × 10^3^	426.978 × 10^3^	423.255 × 10^3^	79.432 × 10^3^	716.199 × 10^3^
70%	MAE	0.787	0.787	**0.258**	0.555	0.555	0.648	1.053	0.743
	RMSE	1.008	1.006	**0.601**	0.846	0.889	0.976	1.341	1.062
	Running time	4.37	4.742	812.783 × 10^3^	1.948 × 10^3^	343.796 × 10^3^	425.170 × 10^3^	78.559 × 10^3^	737.124 × 10^3^
80%	MAE	0.787	0.787	**0.441**	0.632	0.632	0.689	1.05	0.77
	RMSE	1.009	1.009	**0.843**	0.904	0.953	1.028	1.323	1.089
	Running time	3.654	3.974	941.832 × 10^3^	2.222 × 10^3^	391.646 × 10^3^	364.483 × 10^3^	80.943 × 10^3^	610.558 × 10^3^

**Table 4:** Experimental results of different methods in Beijing air quality dataset.

Missing rate	Measures	Mean	Median	kNNI	MICE	SAITS	BRITS	MRNN	Transformer
10%	MAE	0.733	0.724	0.322	0.154	**0.142**	0.161	0.764	0.373
	RMSE	1.031	1.042	0.604	**0.415**	0.417	0.45	1.046	0.649
	Running time	10.169	16.291	59.422 × 10^3^	276.250 × 10^3^	403.224 × 10^3^	1004.883 × 10^3^	160.653 × 10^3^	781.020 × 10^3^
20%	MAE	0.734	0.724	0.323	0.16	**0.148**	0.172	0.786	0.383
	RMSE	1.036	1.047	0.606	**0.414**	0.427	0.463	1.06	0.668
	Running time	13.476	20.383	102.156 × 10^3^	275.906 × 10^3^	417.101 × 10^3^	1013.907 × 10^3^	157.515 × 10^3^	767.878 × 10^3^
30%	MAE	0.734	0.723	0.325	0.173	**0.158**	0.188	0.799	0.403
	RMSE	1.038	1.046	0.62	**0.436**	0.456	0.49	1.076	0.697
	Running time	15.441	20.525	163.666 × 10^3^	279.776 × 10^3^	403.919 × 10^3^	1014.184 × 10^3^	156.793 × 10^3^	592.957 × 10^3^
40%	MAE	0.734	0.724	0.327	0.194	**0.17**	0.206	0.805	0.419
	RMSE	1.034	1.047	0.626	**0.454**	0.465	0.497	1.085	0.719
	Running time	17.046	21.485	316.326 × 10^3^	285.746 × 10^3^	415.838 × 10^3^	1013.742 × 10^3^	157.472 × 10^3^	721.241 × 10^3^
50%	MAE	0.734	0.724	0.33	0.227	**0.191**	0.231	0.834	0.449
	RMSE	1.034	1.046	0.635	0.494	**0.491**	0.52	1.104	0.76
	Running time	17.893	21.454	410.571 × 10^3^	290.209 × 10^3^	416.802 × 10^3^	1010.217 × 10^3^	157.120 × 10^3^	683.609 × 10^3^
60%	MAE	0.734	0.724	0.333	0.278	**0.223**	0.266	0.856	0.486
	RMSE	1.038	1.046	0.644	0.541	**0.529**	0.553	1.12	0.802
	Running time	17.571	20.29	499.211 × 10^3^	292.439 × 10^3^	389.097 × 10^3^	1008.914 × 10^3^	156.501 × 10^3^	673.149 × 10^3^
70%	MAE	0.734	0.724	0.338	0.351	**0.274**	0.32	0.855	0.541
	RMSE	1.037	1.045	0.652	0.62	**0.588**	0.614	1.129	0.859
	Running time	16.004	18.469	588.854 × 10^3^	285.509 × 10^3^	366.107 × 10^3^	939.164 × 10^3^	154.678 × 10^3^	538.320 × 10^3^
80%	MAE	0.734	0.724	**0.347**	0.45	0.349	0.405	0.885	0.592
	RMSE	1.035	1.046	**0.664**	0.729	0.674	0.707	1.148	0.929
	Running time	13.459	15.465	677.042 × 10^3^	284.270 × 10^3^	259.380 × 10^3^	945.751 × 10^3^	154.827 × 10^3^	584.431 × 10^3^

**Table 5:** Experimental results of various methods on Northern Taiwan air quality dataset.

Missing rate	Measures	Mean	Median	kNNI	MICE	SAITS	BRITS	MRNN	Transformer
10%	MAE	0.638	0.624	0.269	0.188	**0.121**	0.136	0.609	0.355
	RMSE	0.91	0.946	0.447	0.401	**0.284**	0.345	0.855	0.566
	Running time	7.803	11.267	57.845 × 10^3^	2780.284 × 10^3^	4223.126 × 10^3^	5403.038 × 10^3^	954.973 × 10^3^	5251.405 × 10^3^
20%	MAE	0.638	0.622	0.27	0.244	**0.129**	0.154	0.649	0.369
	RMSE	0.928	0.933	0.454	0.489	**0.284**	0.362	0.901	0.588
	Running time	9.213	12.109	70.431 × 10^3^	2886.701 × 10^3^	4541.345 × 10^3^	5864.396 × 10^3^	994.876 × 10^3^	5790.410 × 10^3^
30%	MAE	0.638	0.624	0.273	0.3	**0.142**	0.172	0.664	0.394
	RMSE	0.928	0.943	0.457	0.563	**0.307**	0.374	0.906	0.626
	Running time	9.611	11.615	89.274 × 10^3^	2792.366 × 10^3^	4712.072 × 10^3^	5474.605 × 10^3^	958.010 × 10^3^	4429.989 × 10^3^
40%	MAE	0.638	0.623	0.276	0.356	**0.162**	0.194	0.671	0.418
	RMSE	0.925	0.934	0.462	0.64	**0.328**	0.407	0.924	0.666
	Running time	10.947	12.591	108.011 × 10^3^	2730.357 × 10^3^	3042.988 × 10^3^	2008.689 × 10^3^	319.524 × 10^3^	1424.196 × 10^3^
50%	MAE	0.638	0.623	0.28	0.405	**0.186**	0.218	0.696	0.439
	RMSE	0.919	0.938	0.47	0.698	**0.371**	0.43	0.943	0.694
	Running time	10.408	12.302	125.864 × 10^3^	2520.027 × 10^3^	786.227 × 10^3^	1263.780 × 10^3^	337.368 × 10^3^	3507.468 × 10^3^
60%	MAE	0.637	0.623	0.287	0.453	**0.229**	0.249	0.696	0.477
	RMSE	0.921	0.935	0.489	0.757	**0.437**	0.475	0.95	0.743
	Running time	9.133	10.76	164.385 × 10^3^	2588.497 × 10^3^	4882.456 × 10^3^	3001.518 × 10^3^	1006.466 × 10^3^	3295.655 × 10^3^
70%	MAE	0.638	0.623	0.3	0.497	**0.284**	0.29	0.715	0.52
	RMSE	0.922	0.937	**0.516**	0.799	0.521	0.527	0.965	0.805
	Running time	8.843	10.241	157.565 × 10^3^	2809.081 × 10^3^	1578.050 × 10^3^	3672.816 × 10^3^	772.596 × 10^3^	3139.007 × 10^3^
80%	MAE	0.638	0.623	**0.327**	0.542	0.365	0.351	0.713	0.587
	RMSE	0.923	0.934	**0.57**	0.841	0.636	0.605	0.966	0.872
	Running time	7.299	8.234	172.593 × 10^3^	2946.701 × 10^3^	1117.453 × 10^3^	2945.753 × 10^3^	437.275 × 10^3^	1800.182 × 10^3^

**Table 6:** Experimental results of different methods on Dalat (Vietnam) air quality dataset.

Missing rate	Measures	Mean	Median	kNNI	MICE	SAITS	BRITS	MRNN	Transformer
10%	MAE	0.858	0.835	0.267	**0.077**	0.135	0.176	0.599	0.576
	RMSE	1.377	1.738	0.885	**0.218**	0.278	0.721	0.859	1.537
	Running time	0.667	0.936	498.794	557.341 × 10^3^	253.319 × 10^3^	464.648 × 10^3^	209.721 × 10^3^	419.065 × 10^3^
20%	MAE	0.853	0.83	0.269	**0.1**	0.153	0.192	0.638	0.602
	RMSE	1.06	1.516	1.016	0.797	0.83	**0.627**	1.306	0.919
	Running time	0.756	0.99	474.305	529.891 × 10^3^	228.444 × 10^3^	478.094 × 10^3^	187.910 × 10^3^	368.887 × 10^3^
30%	MAE	0.86	0.833	0.264	**0.129**	0.172	0.223	0.663	0.587
	RMSE	1.517	1.716	**0.723**	1.738	1.375	1.002	1.544	1.059
	Running time	0.816	0.995	513.838	536.058 × 10^3^	247.239 × 10^3^	500.513 × 10^3^	221.191 × 10^3^	406.046 × 10^3^

**Table 7:** Experimental results of different methods on Cau Giay (Vietnam) air quality dataset.

Missing rate	Measures	Mean	Median	kNNI	MICE	SAITS	BRITS	MRNN	Transformer
10%	MAE	0.883	0.833	0.618	**0.241**	0.289	0.337	0.782	0.755
	RMSE	1.425	1.336	1.19	0.808	**0.757**	0.911	1.336	1.312
	Running time	0.379	0.933	191.682	71.192 × 10^3^	7.177 × 10^3^	17.692 × 10^3^	10.186 × 10^3^	13.002 × 10^3^
20%	MAE	0.882	0.837	0.617	**0.255**	0.303	0.366	0.792	0.77
	RMSE	1.443	1.485	1.121	1.119	**0.873**	1.018	1.345	1.393
	Running time	0.497	1.035	201.621	70.839 × 10^3^	7.833 × 10^3^	17.299 × 10^3^	10.006 × 10^3^	13.314 × 10^3^
30%	MAE	0.876	0.835	0.618	**0.267**	0.335	0.398	0.811	0.776
	RMSE	1.388	1.401	1.227	1.107	**0.929**	1.045	1.398	1.352
	Running time	0.605	0.948	217.432	71.224 × 10^3^	7.452 × 10^3^	17.223 × 10^3^	9.537 × 10^3^	10.976 × 10^3^
40%	MAE	0.88	0.837	0.617	**0.271**	0.372	0.436	0.81	0.796
	RMSE	1.425	1.456	1.214	**0.984**	1.057	1.016	1.422	1.406
	Running time	0.629	0.781	237.612	71.421 × 10^3^	7.142 × 10^3^	17.180 × 10^3^	9.291 × 10^3^	12.545 × 10^3^
50%	MAE	0.878	0.832	0.618	**0.286**	0.408	0.476	0.814	0.788
	RMSE	1.417	1.39	1.196	1.039	**0.986**	1.055	1.361	1.409
	Running time	0.662	0.793	256.678	71.199 × 10^3^	7.140 × 10^3^	17.157 × 10^3^	9.523 × 10^3^	12.554 × 10^3^
60%	MAE	0.878	0.834	0.617	**0.31**	0.454	0.533	0.833	0.785
	RMSE	1.429	1.424	1.111	1.046	**0.986**	1.075	1.381	1.409
	Running time	0.65	0.744	275.756	70.847 × 10^3^	7.527 × 10^3^	17.116 × 10^3^	8.092 × 10^3^	12.350 × 10^3^
70%	MAE	0.879	0.834	0.619	**0.35**	0.51	0.599	0.827	0.805
	RMSE	1.428	1.442	1.158	**1.081**	1.099	1.221	1.411	1.45
	Running time	0.597	0.669	295.062	71.109 × 10^3^	7.439 × 10^3^	17.134 × 10^3^	7.451 × 10^3^	14.893 × 10^3^
80%	MAE	0.879	0.833	0.625	**0.424**	0.578	0.666	0.854	0.81
	RMSE	1.416	1.43	1.192	**1.07**	1.158	1.264	1.434	1.437
	Running time	0.509	0.582	313.038	71.527 × 10^3^	6.826 × 10^3^	17.133 × 10^3^	5.516 × 10^3^	13.048 × 10^3^

**Table 8:** Experimental results of different methods on Minh Khai District (Vietnam) air quality dataset.

Missing rate	Measures	Mean	Median	kNNI	MICE	SAITS	BRITS	MRNN	Transformer
10%	MAE	1.104	1.024	0.786	**0.338**	0.411	0.489	0.951	0.902
	RMSE	1.517	1.6	1.243	**0.8**	0.85	0.966	1.459	1.426
	Running time	0.608	0.931	202.255	99.009 × 10^3^	8.554 × 10^3^	19.048 × 10^3^	11.317 × 10^3^	13.539 × 10^3^
20%	MAE	1.1	1.022	0.783	**0.352**	0.436	0.512	0.939	0.89
	RMSE	1.512	1.596	1.242	**0.807**	0.9	0.964	1.45	1.406
	Running time	0.73	0.838	209.737	122.288 × 10^3^	8.806 × 10^3^	18.828 × 10^3^	11.271 × 10^3^	12.583 × 10^3^
30%	MAE	1.104	1.025	0.785	**0.365**	0.47	0.54	0.968	0.918
	RMSE	1.521	1.601	1.242	**0.837**	0.923	0.989	1.477	1.438
	Running time	0.83	0.828	228.633	375.988 × 10^3^	9.114 × 10^3^	18.805 × 10^3^	11.048 × 10^3^	12.671 × 10^3^
40%	MAE	1.105	1.023	0.786	**0.387**	0.514	0.583	0.967	0.936
	RMSE	1.531	1.602	1.239	**0.848**	0.995	1.03	1.481	1.469
	Running time	0.908	0.84	250.996	384.147 × 10^3^	8.323 × 10^3^	18.789 × 10^3^	11.146 × 10^3^	14.455 × 10^3^
50%	MAE	1.103	1.026	0.789	**0.41**	0.563	0.631	0.984	0.935
	RMSE	1.525	1.606	1.25	**0.893**	1.049	1.1	1.507	1.479
	Running time	0.759	0.835	272.51	381.077 × 10^3^	8.212 × 10^3^	18.785 × 10^3^	11.155 × 10^3^	16.029 × 10^3^
60%	MAE	1.105	1.024	0.791	**0.442**	0.615	0.679	0.999	0.966
	RMSE	1.527	1.597	1.267	**0.932**	1.112	1.16	1.522	1.533
	Running time	0.692	0.805	294.391	389.190 × 10^3^	8.642 × 10^3^	18.779 × 10^3^	9.922 × 10^3^	13.160 × 10^3^
70%	MAE	1.105	1.024	0.794	**0.494**	0.67	0.745	1.007	1.009
	RMSE	1.526	1.594	1.268	**0.992**	1.183	1.236	1.529	1.587
	Running time	0.636	0.727	314.352	389.794 × 10^3^	9.214 × 10^3^	18.785 × 10^3^	9.133 × 10^3^	10.024 × 10^3^
80%	MAE	1.106	1.022	0.803	**0.577**	0.766	0.838	1.041	1.014
	RMSE	1.526	1.595	1.277	**1.09**	1.307	1.359	1.584	1.583
	Running time	0.546	0.613	335.587	373.516 × 10^3^	7.816 × 10^3^	18.795 × 10^3^	7.257 × 10^3^	10.533 × 10^3^

### 6.2 Results on different datasets

#### 6.2.1 Frankfurt dataset

Table 3 presents the results of the traditional imputation method compared with other imputation methods regarding the experiment accuracy and running time on the Frankfurt air quality dataset. One can see that when the missing rate of the dataset increases from 10% to 80%, Mean and Median methods have an almost constant MAE error (fluctuating around 0.787–0.788); the MRNN model gives the highest error (greater than 0.904). Moreover, the Transformer model has an MAE error from 0.651 to 0.77; the kNNI model alone has the smallest MAE and RMSE errors among the remaining machine learning models, such as MICE, even lower than the currently used neural-network models, such as SAITS and BRITS. In general, in this dataset, the MAE and RMSE errors of the kNNI model both give the lowest and most stable results among the remaining missing data models when the missing rate of the original data is 0%, and the artificial missing data rate gradually increases from 10% to 80%. As depicted in Fig 2c, it is worth noting that the running time of the models used in this dataset mainly increases when the missing rate of the dataset changes from 10% to 80%. On the other hand, compared to traditional models or basic machine learning models, although models based on neural networks have a long calculation time, MRNN gives relatively positive results and is the most effective among the models using neural networks in terms of Mean, Median, and MICE.

On the other hand, when the original dataset is not missing and the data size is larger than one million records (for example, Frankfurt air quality data), kNNI is considered the model that gives the best results with an artificial missing rate of 10%–80% and time execution time gradually increases from 62.684 × 10^3^ milliseconds to 941.832 × 10^3^ milliseconds, followed by SAITS with computation time decreasing from 843.803 × 10^3^ milliseconds to 391.645 × 10^3^ milliseconds.

#### 6.2.2 Beijing dataset

Table 4 depicts the results of imputation methods compared with other imputation methods on the Beijing air quality dataset. In this dataset, the SAITS model also gives the lowest MAE measure compared to other machine learning models; the model error varies from 0.142 to 0.349, followed by BRITS. Meanwhile, the traditional data-filling models vary from 0.724 to 0.885 as the missing ratio gradually increases from 10% to 80%. On the other hand, the MAE error of the SAITS model when the artificial missing rate of data changes from 10% to 40% is lower than that of the MICE model; on the contrary, the RMSE error of MICE is lower than that of SAITS, and the lowest in the remaining used models such as BRITS, kNNI, Transformer, Mean, Median, and MRNN. The artificial missing rate of data ranges from 50% to 70%, the MAE and RMSE errors of the SAITS model are stable again, and the experimental results obtained are the smallest among the models. The remaining models give a relatively large error, with MRNN having the largest error. When the missing data rate is at 80%, the kNNI model gives the best MAE and RMSE errors compared to traditional data filling or machine learning models. In addition, Fig 3c shows the computational time of models such as Median, MRNN, MICE, SAITS, and BRITS remains almost constant when the missing rate increases from 10% to 80%. Next, kNNI is a model with large fluctuations in calculation time, gradually increasing as the missing rate increases. Moreover, the calculation time of the Transformer gradually decreases and changes sharply as the missing ratio increases. Compared to traditional machine learning models, MRNN has the most stable and fastest calculation time compared to the remaining models in this dataset.

#### 6.2.3 Taiwan dataset

Table 5 shows that the MAE error between traditional models and current models using neural networks grows larger as the missing rate of input temporal data increases on the Northern Taiwan air quality dataset. Specifically, SAITS is the model with the lowest error (ranging from 0.121 to 0.284), followed by BRITS (error only from 0.136 to 0.29) in this data. Meanwhile, methods such as Mean, Median, kNNI, or MICE give errors when filling in missing values that deviate greatly from the original value. Besides, when the artificial missing rate of the data changes from 10% to 60%, the MAE and RMSE errors of the SAITS model give the lowest results. However, when the missing data rate reaches 70%, the SAITS model’s MAE error is the smallest, but the RMSE error is higher than that of the kNNI model. When the missing rate is from 80%, the MAE and RMSE error results of kNNI are the lowest among the models, followed by BRITS.

On the one hand, in Fig 4c, we can see the computational time of machine learning models like kNNI is the smallest after traditional missing data filling models like Mean and Median. Besides, MRNN is the model with the least computational time among machine learning models, followed by MICE. The remaining models have fluctuating and irregular calculation times, the highest when the missing data rate is 10%–30%, and the lowest when the missing data rate is 40%–50%. By comparing the performance of the Mean, Median, kNNI, MICE, SAITS, BRITS, MRNN, and Transformer models, we see that SAITS is the best missing data imputation model on Northern Taiwan and Beijing air quality dataset with an artificial missing rate under 70%. One can see that when the original missing rate of the dataset is less than 30% (or 10%). The dataset only has a few hundred thousand records. SAITS seems to be the model with the lowest error, and model execution time also gradually decreased (from 4223.126 × 10^3^ milliseconds to 1117.453 × 10^3^ milliseconds with the Northern Taiwan dataset and from 403.223 × 10^3^ milliseconds to 259.380 × 10^3^ milliseconds with the Beijing dataset) as the missing rate of the dataset increased.

#### 6.2.4 Dalat dataset

Table 6 presents similar experimental results on the Dalat air quality dataset [51]. In this table, traditional methods such as Mean and Median give a constant MAE measure (about 0.83–0.86) when the missing rate of data changes from 10% to 30% and almost the result of these measures is the largest compared to the remaining missing data filling models. Meanwhile, the neural network models used in this dataset, such as SAITS and BRITS, give optimal results, which are not much different from traditional machine learning models such as kNNI and MICE (for MAE measurement, the shortest). However, when the artificial missingness ratio of the data is at 10%, MICE gives relatively low MAE and RMSE errors among the models. Furthermore, when increasing the artificial missing rate to 20%, although the MAE error of MICE is the lowest, the RMSE error of the BRITS model is the smallest. Next, when continuing to increase the artificial missing rate of the model to 30%, the experimental results, MICE is the model with the smallest MAE, and kNNI is the model with the lowest RMSE of all. On the other hand, the calculation time of most models increases when the data’s artificial missing rate increases. Accordingly, MRNN is the model with the fastest computation time, followed by SAITS, Transformer, BRITS, and MICE in Fig 5c.

#### 6.2.5 Cau Giay dataset

The experimental results on the Cau Giay District air quality dataset [49] are presented in Table 7. One can see that the MAE errors of MICE showed the best results among the used models (only from 0.241–0.424) when the artificial missing rate of the data gradually increased from 10% to 80%. The second best is SAITS (from 0.289–0.578), and the third one is BRITS (from 0.337–0.666). However, the RMSE error of SAITS is the lowest with artificial missing rates of 10%–30% and 50%–60%. Meanwhile, when the missing rate is 40% and increases to 70%–80%, MICE almost always gives relatively good results compared to the remaining models. Besides, the MAE and RMSE errors of the models become larger when the missing rate changes from 10% to 80%, especially the MAE and RMSE errors of the two methods Mean and Median are large, only fluctuating around 0.8. In addition, the running time of machine learning and neural network models is significantly slower than Mean imputation (0.379–0.509 milliseconds) and Median imputation (0.933–0.582 milliseconds). Also, the slowest is MICE, with a relatively large running time, ranging from 71.192 × 10^3^ to 71.527 × 10^3^ milliseconds.

#### 6.2.6 Minh Khai dataset

Table 8 presents experimental results on the Minh Khai air quality dataset [49]. Although the experimental time of MICE is the largest, the model gives the most optimal MAE and RMSE error results among the models used, followed by SAITS, BRITS, kNNI, and Transformer. The MAE and RMSE errors of the Mean and Median methods hardly change much when increasing the missing data rate from 10% to 80%; MAE and RMSE errors of the remaining models gradually increase as the missing data rate increases.

Furthermore, one can also see that when the original missing rate of data is less than 5%, MICE is the method that gives the most optimal results among the missing data imputation methods used in this article (specifically with the Cau Giay district and Minh Khai district air quality dataset). Besides, the running time of MICE is high and increases the fastest when the missing rate of data gradually increases with the Minh Khai dataset. On the other hand, the Cau Giay dataset does not change much over time. However, when considering the performance of filling in missing values using neural networks, SAITS is the model with the most optimal performance, followed by BRITS. We knew that Transformer is a deep-learning model designed to solve many problems. However, in this study, we can see that Transformer hardly promotes its strengths, and experimental results on different data all give much larger MAE and RMSE errors than SAITS and BRITS.

### 6.3 The impact of different numbers of layers

Based on the experimental results presented above, although MICE gives a better MAE measure than SAITS in some cases (specifically in datasets in Vietnam), the running time of MICE is many times longer than that of SAITS. Therefore, we propose SAITS as the model to fill in missing values for the air quality data for the multivariate time series of those tested in this paper. We now perform another test and compare the results when performing missing data filling on air quality data of SAITS with the Transformer model with the different number of layers cases (i.e., two layers, four layers, and six layers) and the BRITS model. We then propose the best model to fill in the last missing value before predicting air quality for the following year.

**Fig 8:**
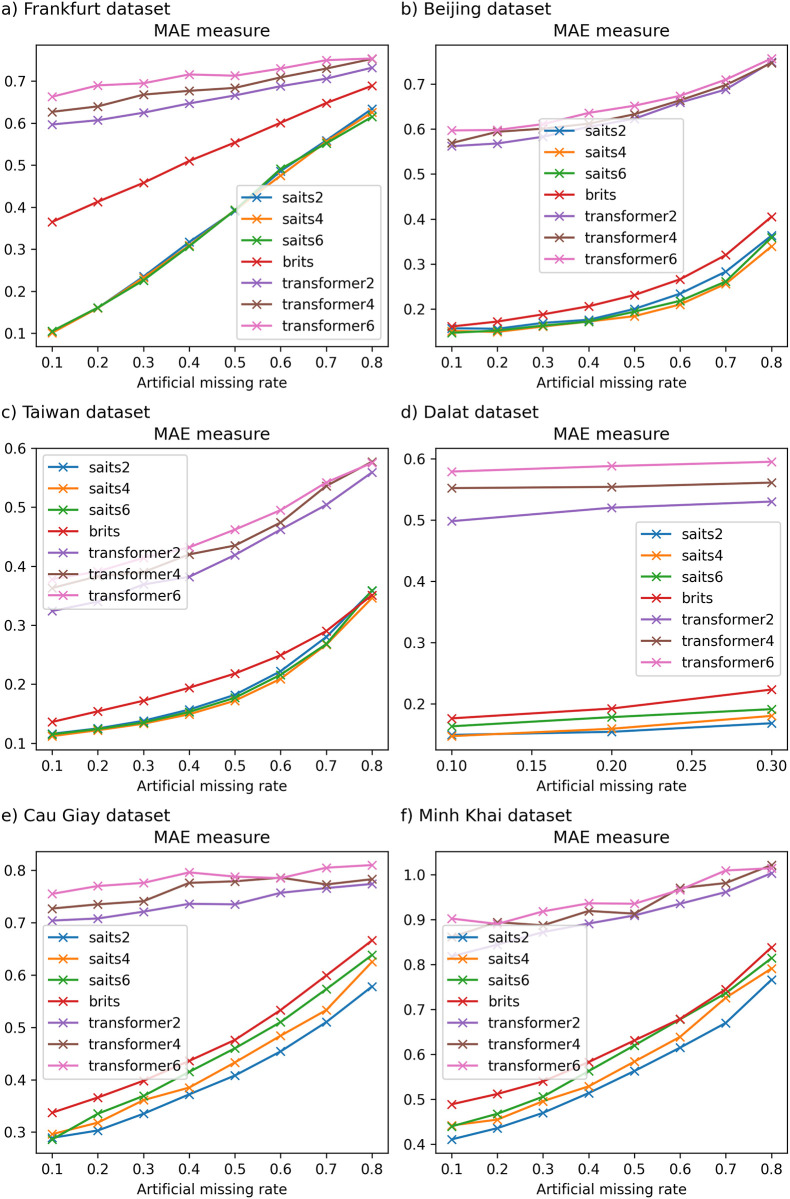
Evaluating MAE of the SAITS method with the different number of layers on various datasets.

**Fig 9:**
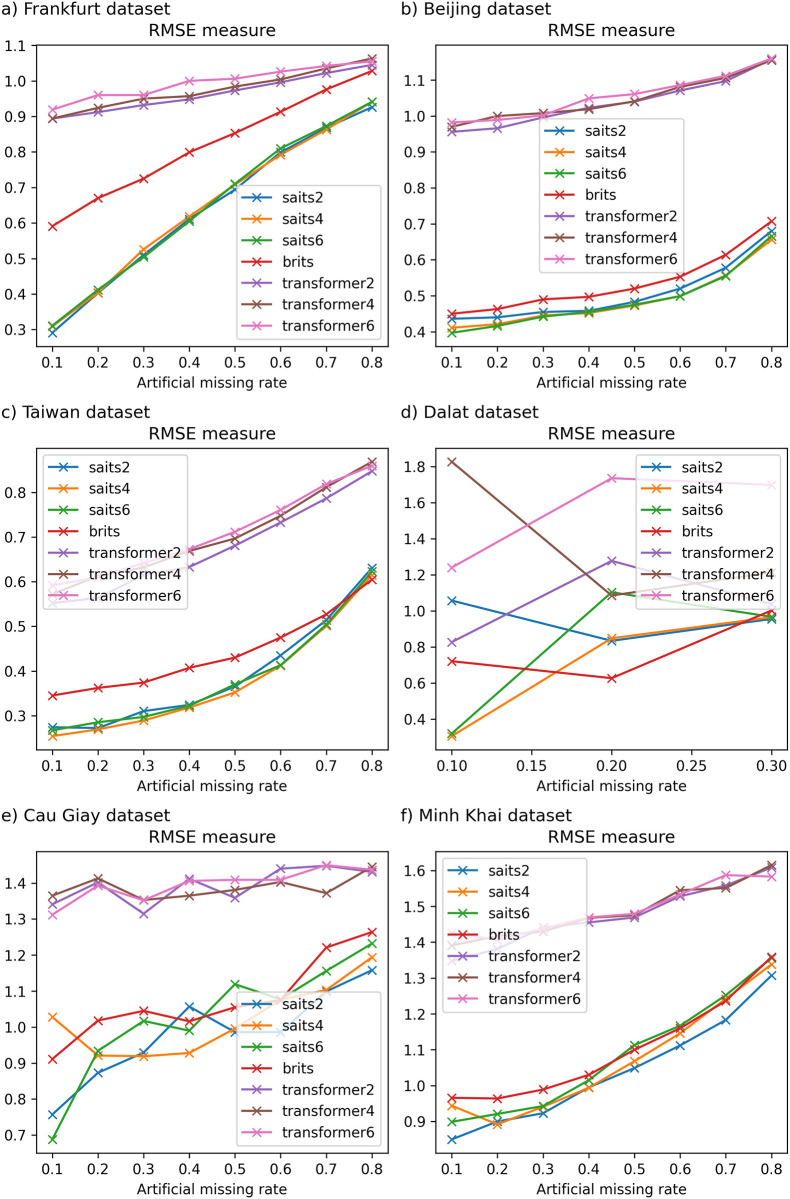
Evaluating RMSE of the SAITS method with the different number of layers on various datasets.

**Fig 10:**
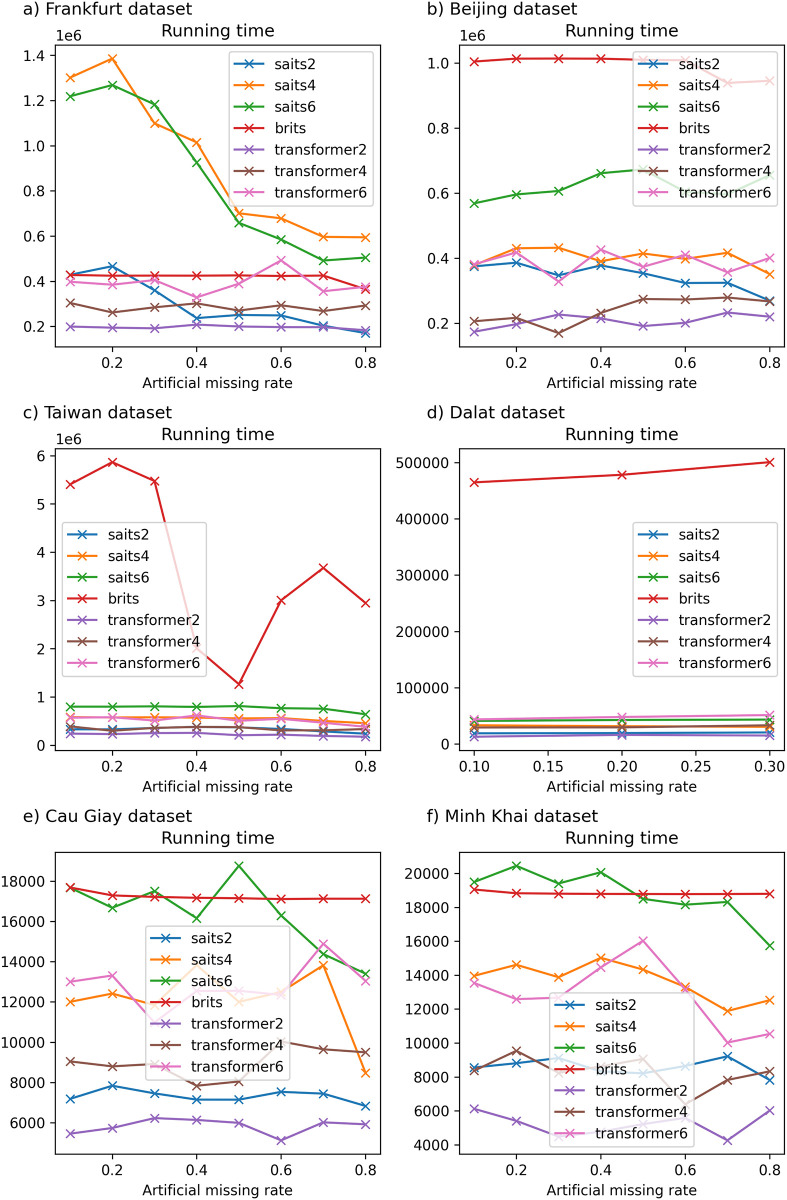
Evaluating the running time of the SAITS method with the different number of layers on various datasets.

The result of evaluating Transformer and SAITS with two-layer, four-layer, and six-layer for both datasets is presented accordingly in Fig 8. From there, one can see that SAITS with two, four, and six layers do not show as clearly as Transformer with two, four, and six layers in the six datasets, including Frankfurt, Beijing, and Taiwan. However, for the Dalat, Cau Giay, and Minh Khai datasets, we can see the results of both SAITS and Transformer with two, four, and six layers clearly shown. As a result, SAITS with two layers performs better than Transformers with two, four, and six layers. Besides, the RMSE performance of SAITS and Transformer with layers of all datasets in Fig 9 is unclear and changes frequently. In contrast, the running time of SAITS and Transformer with two layers in Fig 10 for both datasets is also a more effective model with four layers and six layers.

### 6.4 Predict air quality in Vietnam combined AQI indexes

Based on the results and evaluating the performance of the methods with different numbers of layers above, we propose the SAITS model with two layers as the most optimal missing value estimation technique with a small sample size on the three air quality datasets in Vietnam.

We started predicting factors affecting air pollution in the following 24 hours based on Vector Auto-Regression (VAR) and related features. Next, we analyzed daily air pollution levels across countries on three datasets in Vietnam, which were mentioned based on the Air Quality Index (AQI). The AQI level of each pollutant is calculated according to the instruction (Available on: https://www.airnow.gov/sites/default/files/2020-05/aqi-technical-assistance-document-sept2018.pdf) and divided into seven levels (i.e., Good, Moderate, Unhealthy for Sensitive Groups, Unhealthy, Very Unhealthy, Hazardous, and Extreme Hazardous). It only includes six air pollutants (i.e., *PM*2.5, *PM*10, *CO*, *NO*_2_, *SO*_2_, and *O*_3_) that are required to predict their values and related AQI levels. This index tells us how clean or polluted the air is and what it means to human health. The higher the AQI index value, the greater the level of air pollution and the more negative impact it has on human health.

Additionally, we also perform the outlier detection for the datasets using Mahalanobis distance, where points with large Mahalanobis distance (greater than 97.5%) are considered to be outliers. However, we didn’t remove the outliers from the datasets because deleting them will make the data become irregularly sampled time series data, and VAR is not designed for that type of data.

We analyze indexes such as *SO*_2_; *CO*; *PM*10; *PM*2.5; *O*_3_ recorded by sensors in Dalat, Vietnam. The concentration of *PM*2.5 collected at some stations is large (ranging from 50–100*μg*/*m*^3^), but the air quality collected from stations in this place is at an acceptable average level, some stations exceeding the threshold range from 100–106*μg*/*m*^3^ but at a poor level and affecting sensitive groups of people. (Similar to the concentration of fine particles *PM*10). Meanwhile, the levels of *CO*, *SO*_2_, and *O*_3_ in the air are low, only from 1.09–10.7*μg*/*m*^3^, completely at a good level and do not have much impact on human health. In general, from the end of 2019 to the beginning of 2020, the concentration of these indicators increased and posed serious harm to human health. Besides, some other factors can interfere with the fluctuation of air pollution in Dalat City, known as the city of thousands of flowers, and attract many people, especially young people, to visit and relax during festivals or on the weekend. Next, Dalat is geographically located in a mountain valley and has cool weather that may impact air pollution and some activities.

Next, we also analyzed factors such as *PM*10, *PM*2_5, *CO*, *SO*_2_, *O*_3_ recorded by two districts of Cau Giay and Minh Khai in Hanoi, Vietnam. Fine dust concentrations *PM*10 and *PM*2.5 in the Cau Giay district are mostly at moderate levels according to the AQI categories table, and they do not cause serious effects on human health. However, on some days when the concentration of these indicators is recorded at a high level, greater than 100*μg*/*m*^3^, and even on some days, the concentration of fine dust in some time frames exceeds 300*μg*/*m*^3^, seriously affecting the health of the people of the Capital. In addition, the number of motorbikes and vehicles circulating in Hanoi is quite large, which is also the cause of air pollution here; *CO* concentration is greater than 1000*μg*/*m*^3^. For example, from 22/2/2023-23/2/2023, *CO* concentration was recorded above 10, 000*μg*/*m*^3^ at an alarming level. In addition, the concentration of *SO*_2_ is relatively low, possibly because this area does not have many manufacturing plants, so the amount of toxic chemicals such as *SO*_2_ released into the environment is small, at a good level, and does not affect the human health. From the end of 03/2019 to mid-04/2019, the concentration of *O*_3_ gradually increased, from 100–350*μg*/*m*^3^, changing from a level that is not harmful to human health to a seriously harmful level. Besides, the concentration of fine dust particles *PM*10, *PM*2.5 in the air recorded in the Minh Khai district dataset is also quite high, which is not good for human health. Some days, the fine dust concentration of these particles exceeds 479*μg*/*m*^3^ and 255*μg*/*m*^3^, respectively *PM*10 and *PM*2.5. Furthermore, the amount of *SO*_2_ and *O*_3_ in the Minh Khai district dataset is quite low, at a normal level according to the AQI categories scale, so it does not affect human health. However, the concentration of *CO* in the air is very high; most days, the concentration is recorded to be greater than 1000*μg*/*m*^3^, which is considered “Exceeding AQI.” Recommendations for hazard classification should be implemented. In summary, Hanoi is known to domestic and foreign friends as the Capital of Vietnam, a place to work and welcome heads of state. Based on the air pollution problem in the Cau Giay and Minh Khai districts, we need to take many measures to reduce air pollution and improve the environment to attract tourists, creating economic development and international cooperation conditions.

## 7 Discussion

There are many estimation techniques to handle missing data. A lot of them focus on the missing values of the time series. However, these techniques might not be able to capture time information and produce reliable imputation results if timestamps are missing. Therefore, expanding on current techniques to impute missing timestamps may be suitable for handling such situations. Research on deep learning modeling is a being cared area. Numerous novel deep-learning models with practical applications have been proposed in recent years. There is a growing number of research papers on deep learning for imputing missing data, and these new approaches seem promising. However, in practical applications, the validity and strength of these models must be carefully assessed. In many cases, reliable and reproducible code is frequently unavailable or incomplete. Compared to conventional statistical methods, the number of hyperparameters for deep learning models typically requires much more tuning. In some applications, the hyperparameter search on big data may be prohibitively expensive due to the required training time or memory size. However, our research showed that for data with small, moderate, or even large sample sizes (i.e., when the sample size is less than 30,000), the stability and convergence of the deep learning models needed to be revised.

Numerous factors, including the sample size of the data, the distribution of the variables, the number of missing values in the data, the correlation structure of the data, and potential missingness mechanisms, affect how effective different missing data imputation techniques are.

While this work presents a thorough investigation of time series imputation techniques and provides practical implications for practitioners, it also has some limitations. For example, different types of geographic locations, such as mountains and plains, may affect air quality. Collecting more datasets and examining the patterns for various types of geographic locations may help draw out common patterns and insights to improve the imputation quality. However, due to limited data available, we have not achieved that goal yet. In addition, this work so far has only concentrated on examining the effect of missing data for the missing at random pattern. However, it is also possible that air quality data is missing, not at random. These will be topics for our future research.

## 8 Conclusions

We have presented an investigation of the impact of data imputation techniques on the air quality prediction problem. In general, SAITS gives the lowest error, and the difference in running time is negligible compared to the missing value imputation efficiency that SAITS provides when the missing rate increases. Besides, BRITS is the model that gives the second-best error among deep learning models, only after SAITS. kNNI running time can increase significantly as the missing rate increases. However, this may not be true for other methods. Also, kNNI proves itself to remain a promising imputer for the dataset. In addition, for three datasets (Northern Taiwan, Beijing, and Frankfurt) which have a large sample size, at the high missing rate of 80%, kNNI outperforms other techniques, including the state-of-the-art such as SAITS, BRITS, and Transformer. Meanwhile, other cases were varied using limited sample size and ratios of missing data. The experiment results show the conventional method, MICE, outperforms the recently proposed deep learning methods, such as SAITS and BRITS, in these experiments.

The outcomes of this article can open a new direction for predicting air pollution. However, as discussed, it also has some limitations, such as the experiments concentrated only on missingness at random, and the paper has not been able to draw insights by grouping datasets based on geological characteristics due to limited data. Therefore, in the future, we will collect more datasets to examine the imputation quality based on types of geographic locations or some other characteristics and consider the imputation effects for nonrandomly missing data. Also, we will develop an ensemble technique combining the latest missing techniques and SAITS to enhance the imputation performance. Moreover, the process can integrate the knowledge in a particular domain to support extracting helpful information from datasets [86]. In addition, in the future, we plan to empirically evaluate the performance of imputation techniques for other types of environmental data, such as imbalanced missing data. Last but not least, we want to investigate if methods to combine datasets such as ComImp [87] can be used to combine air quality datasets to improve the imputation and prediction quality.
